# Exercise-dependent formation of new junctions that promote STIM1-Orai1 assembly in skeletal muscle

**DOI:** 10.1038/s41598-017-14134-0

**Published:** 2017-10-27

**Authors:** Simona Boncompagni, Antonio Michelucci, Laura Pietrangelo, Robert T. Dirksen, Feliciano Protasi

**Affiliations:** 1 CeSI-Met - Center for Research on Ageing and Translational Medicine, University G. d’Annunzio, Chieti, I-66100, Italy; 2 DNICS - Department of Neuroscience, Imaging and Clinical Sciences, University G. d’Annunzio, Chieti, I-66100 Italy; 3 Department of Pharmacology and Physiology, University of Rochester Medical Center, Rochester, NY 14642 USA; 4 DMSI - Department of Medicine and Aging Science, University G. d’Annunzio, Chieti, I-66100, Italy

## Abstract

Store-operated Ca^2+^ entry (SOCE), a ubiquitous mechanism that allows recovery of Ca^2+^ ions from the extracellular space, has been proposed to limit fatigue during repetitive skeletal muscle activity. However, the subcellular location for SOCE in muscle fibers has not been unequivocally identified. Here we show that exercise drives a significant remodeling of the sarcotubular system to form previously unidentified junctions between the sarcoplasmic reticulum (SR) and transverse-tubules (TTs). We also demonstrate that these new SR-TT junctions contain the molecular machinery that mediate SOCE: stromal interaction molecule-1 (STIM1), which functions as the SR Ca^2+^ sensor, and Orai1, the Ca^2+^-permeable channel in the TT. In addition, EDL muscles isolated from exercised mice exhibit an increased capability of maintaining contractile force during repetitive stimulation in the presence of 2.5 mM extracellular Ca^2+^, compared to muscles from control mice. This functional difference is significantly reduced by either replacement of extracellular Ca^2+^ with Mg^2+^ or the addition of SOCE inhibitors (BTP-2 and 2-APB). We propose that the new SR-TT junctions formed during exercise, and that contain STIM1 and Orai1, function as *Ca*^*2+*^ *Entry Units (CEUs)*, structures that provide a pathway to rapidly recover Ca^2+^ ions from the extracellular space during repetitive muscle activity.

## Introduction

Store-operated Ca^2+^ entry (SOCE) is a ubiquitous Ca^2+^ entry mechanism, first described in non-excitable cells, that is triggered by depletion of intracellular Ca^2+^ stores (endoplasmic/sarcoplasmic reticulum, respectively ER and SR)^1,2^. SOCE is coordinated by the communication between: a) stromal interaction molecule-1 (STIM1), which acts as the Ca^2+^ sensor in the ER lumen^3,4,5^, and b) Orai1, the Ca^2+^-permeable channel in the plasma membrane (PM)^6,7^. The mechanism for Orai1 activation in non-excitable cells involves store depletion causing STIM1 oligomers to aggregate and translocate to form junctions with the PM^8–10^. Aggregated STIM1, in turn, recruits and traps Orai1 channels into these ER-PM junctions, or *puncta*, resulting in the activation of Orai1 channels that mediate entry of Ca^2+^ into the cell from the extracellular space^11^.

SOCE in skeletal muscle^12–14^ is similarly mediated by interactions between STIM1 in the SR and Orai1 channels in the PM^15,16^ and is proposed to limit muscle fatigue during repetitive stimulation^17^. Recently, using constitutive and inducible, muscle-specific Orai1 knockout mice, Carrell and collegues reported that while Orai1 limits muscle fatigue by promoting the growth and maintenance of fatigue-resistant type I fibers, fatigue and endurance exercise were unaltered following post-developmental, muscle-specific ablation of Orai1^18^. Thus, the precise mechanisms by which Orai1 impacts muscle fatigue remain controversial. In addition to its role in healthy muscle, altered SOCE activity is also linked to several distinct forms of muscle dysfunction. For example, a reduction in SOCE activity was proposed to contribute to muscle impairment in aging^19,20^, though this finding was not confirmed by others^21,22^. On the other hand, an increase in Orai1-mediated SOCE exacerbates muscular dystrophy in *mdx* mice^23–26^ and mutations in STIM1 and Orai1 are linked to Tubular Aggregate Myopathy (TAM)^27–31^, an autosomal dominant muscle disease that is clinically characterized by myalgia, cramps and muscle stiffness, with or without proximal muscle weakness. Together, these findings from prior studies suggest that Orai1-mediated SOCE plays an important role in skeletal muscle function and disease.

Experiments in skinned skeletal muscle fibers have provided evidence that SOCE channels are present in transverse tubules (TTs), specialized invaginations of the PM that propagate the action potential into the fiber interior, and activate rapidly following SR Ca^2+^ store depletion^12,32^. These studies suggest that rapid SOCE in the TT system might involve STIM1-Orai1 coupling within the pre-formed SR-TT junctions of the triad, sites of excitation-contraction (EC) coupling also known as Ca^2+^ release units (CRUs)^33,34^. However, to date the precise subcellular sites of STIM1-Orai1 interaction during SOCE in skeletal muscle fibers has not been unequivocally identified. Indeed, given the tight packing of type 1 ryanodine receptor (RyR1) arrays in the terminal SR^33^, there may not be sufficient space within the triad to also accommodate large STIM1 aggregates needed to trap and activate Orai1 channels following store depletion. In addition, the junctional gap width of the triad (~12 nm) is larger than the corresponding gap width (~8 nm) between the ER and PM that accommodates STIM1 aggregates as estimated in non-excitable cells transfected with YFP-STIM1^35^.

Here we used a combination of electron microscopy (EM), immunofluorescence, immunogold, and *ex vivo* muscle contractility techniques to assess STIM1 and Orai1 subcellular localization and skeletal muscle function in adult mice under resting conditions and after a single bout of treadmill exercise. Our results show that exercise drives a significant remodeling of the sarcotubular system within the I band of the sarcomere that results in the formation of previously unidentified junctions between the SR and TTs that contain STIM1 and Orai1 proteins. In addition, this remodeling is accompanied by an increased resistance to fatigue during repetitive high frequency stimulation that is significantly reduced by preventing Ca^2+^ influx following either removal of extracellular Ca^2+^ or addition of SOCE inhibitors.

## Results

### Treadmill exercise drives rearrangement of membranes at the I band into stacks of flat-cisternae

**Figure 1:**
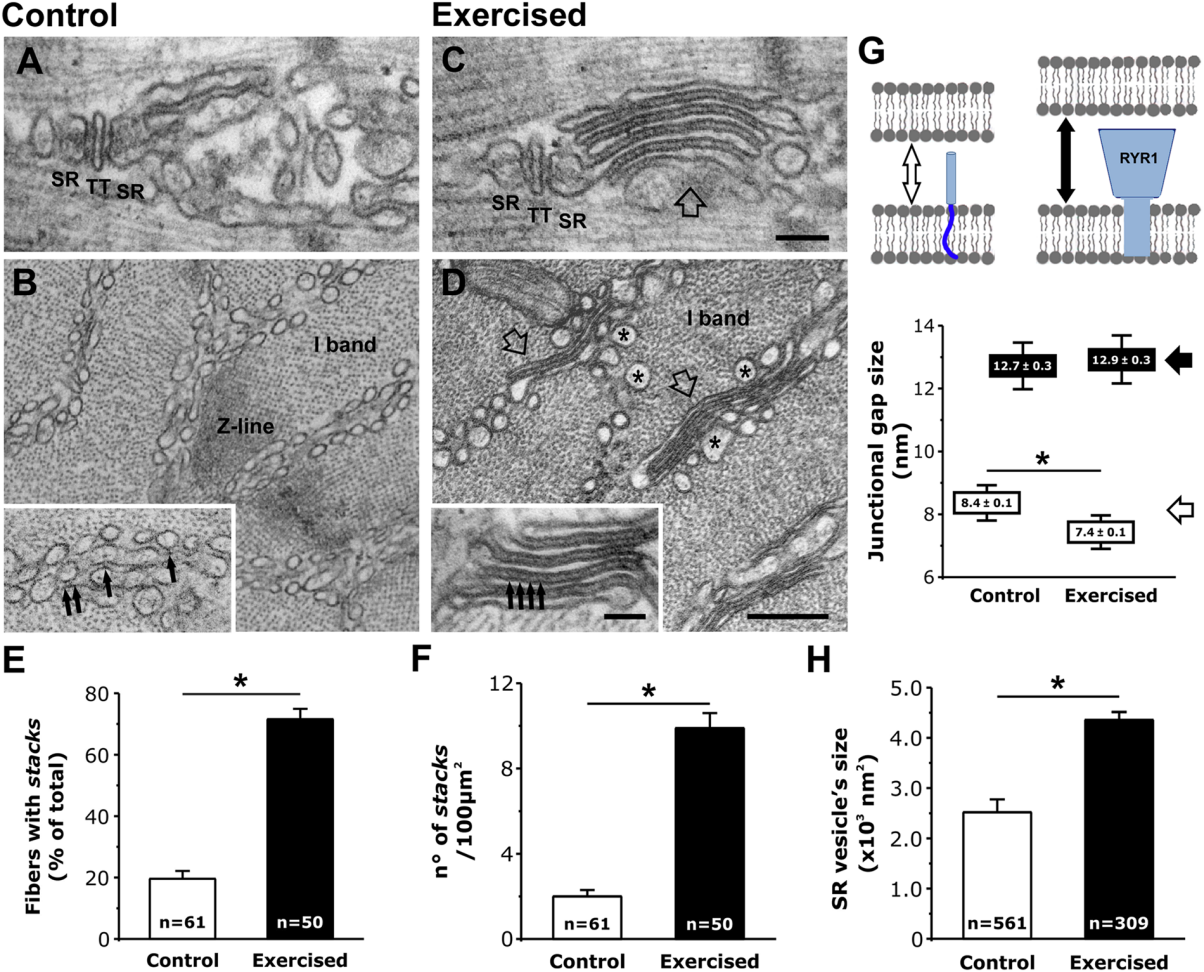
Following exercise, membranes at the I band rearrange into stacks of flat-cisternae. (**A**–**D**) Longitudinal (**A** and **C**) and cross (**B** and **D**) EM sections in proximity of the I bands. Empty arrows in (**C** and **D**) point to newly formed stacks of flat-cisternae; arrows in insets point to strands between SR vesicles (**B**) and flat-cisternae (**D**). (**E** and **F**) Incidence of stacks. *Sample size: control, 3 mice; exercised, 3 mice*. (**G**) Junctional gap size in membrane stacks (empty arrows) and in triads (black arrows) measured as shown in the cartoon. *Samples size: control, 3 mice/76 measurements/21 stacks analyzed; exercised, 3 mice/119 measurements/34 stacks analyzed*. (**H**) Average size of SR vesicles at the I band (some larger ones are marked by asterisks in panel D). *Sample size: control, 3 mice/561 measurements; exercised, 3 mice/309 measurements*. Data are shown as mean ± SEM; *p < 0.01. Numbers in bars (n) indicate the number of fibers analyzed (**E** and **F**) and the number of SR vesicles measured (**H**). Scale bars: (**A** and **C**) (and insets in **B** and **D**), 0.1 μm; (**B**) and (**D**), 0.2 μm.

In mammalian skeletal muscle, the SR is composed of two morphologically and functionally different regions: 1) the junctional SR (also known as SR terminal cisternae) located at the I-A band transition of relaxed sarcomeres, which associates with TTs to form triads (labeled SR-TT-SR in Fig. 1A,C); and 2) the free-SR, which is localized either next to the A band or within the I band (on both sides of Z lines). In EM, the free-SR at the I band is formed by convoluted tubules that appear as multiple layers of vesicles when viewed in cross-sections (Fig. 1B). However, following a single session of treadmill exercise (~1 hour while progressively increasing treadmill speed from 10 m/min to 25 m/min), regions of the free-SR within the I band exhibited a profound remodeling that involved the formation of stacks of flat-cisternae composed of multiple elements (Fig. 1C,D, empty arrows). In Fig. S1, the positioning of these stack of flat-cisternae at the I band (between the triad and the Z line) was more evident. In addition to SR, these stacks also contained TT membranes (see next paragraph). Stacks of flat-cisternae were also occasionally observed in muscle from non-exercised mice, but were significantly more frequent (Fig. 1E,F), larger in size, and formed by more elements (Fig. S2) following treadmill exercise. Table 1 summarizes the number/area of EM section of stacks of flat-cisternae to that of CRUs (i.e. triads) in EDL muscles from control and exercised mice. Under control conditions, EDL muscles contained 73.6 ± 2.1 CRUs/100 μm^2^ and only 2.0 ± 0.3/100 μm^2^ stacks of flat cisternae (ratio of 37:1; Table 1, column C). However, following exercise, the number of membrane stacks increased significantly (Fig. 1E,F; and Table 1, column B), while the number of CRUs remained constant (Table 1, column A). As a result, the ratio between CRUs and stacks of flat-cisternae decreased from 37:1 at rest to 7:1 following exercise (Table 1, column C). Finally, we quantified two additional parameters:

- Small electron-dense strands were present between SR vesicles in control (inset in Fig. 1B) and between flat cisternae following exercise (inset in Fig. 1D). We measured the junctional gap size containing electron-dense strands and compared it with the junctional gap occupied by RyR1-feet in triads (as shown in Fig. 1G, top). The gap size in control samples (measured between SR vesicles where small electron-dense strands were visible) was significantly smaller than that of triads: 8.4 ± 0.1 vs. 12.7 ± 0.3 nm (p < 0.01) (Fig. 1G, bottom). Following exercise, while the junctional gap size in triads remained unaltered (12.9 ± 0.3 nm), the gap size in stacks of flat-cisternae (containing small electron-dense strands) was significantly reduced from 8.4 ± 0.1 to 7.4 ± 0.1 nm (p < 0.01).
- Following exercise, membrane vesicles located near stacks of flat-cisternae (those marked by asterisks in Fig. 1D) were significantly larger than those observed in control muscles, a visual observation supported by direct measurements from EM images (Fig. 1H).

**Table 1:** The number/area of stacks of flat-cisternae, but not that of triads, increases following exercise.

	A	B	C
	No. of CRUs/100 μm^2^	No. of stacks/100 μm^2^	CRUs/stacks ratio
Control	73.6 ± 2.1	2.0 ± 0.3	37/1
Exercised	71.9 ± 1.8	9.9 ± 0.7*	7/1

Columns A and B) number/area of CRUs and of stacks of flat- cisternae. Column C) Ratio between number/area of CRUs and stacks of flat-cisternae. *Sample size: control, 2 mice (14 fibers and 5–10 micrographs/fiber); exercised, 3 mice (21 fibers and 5 micrographs/fiber)*. Data are shown as mean ± SEM; *p < 0.01 vs. control.

### Following exercise, TT extensions at the I band are significantly more frequent and are associated with stacks of flat-cisternae

**Figure 2:**
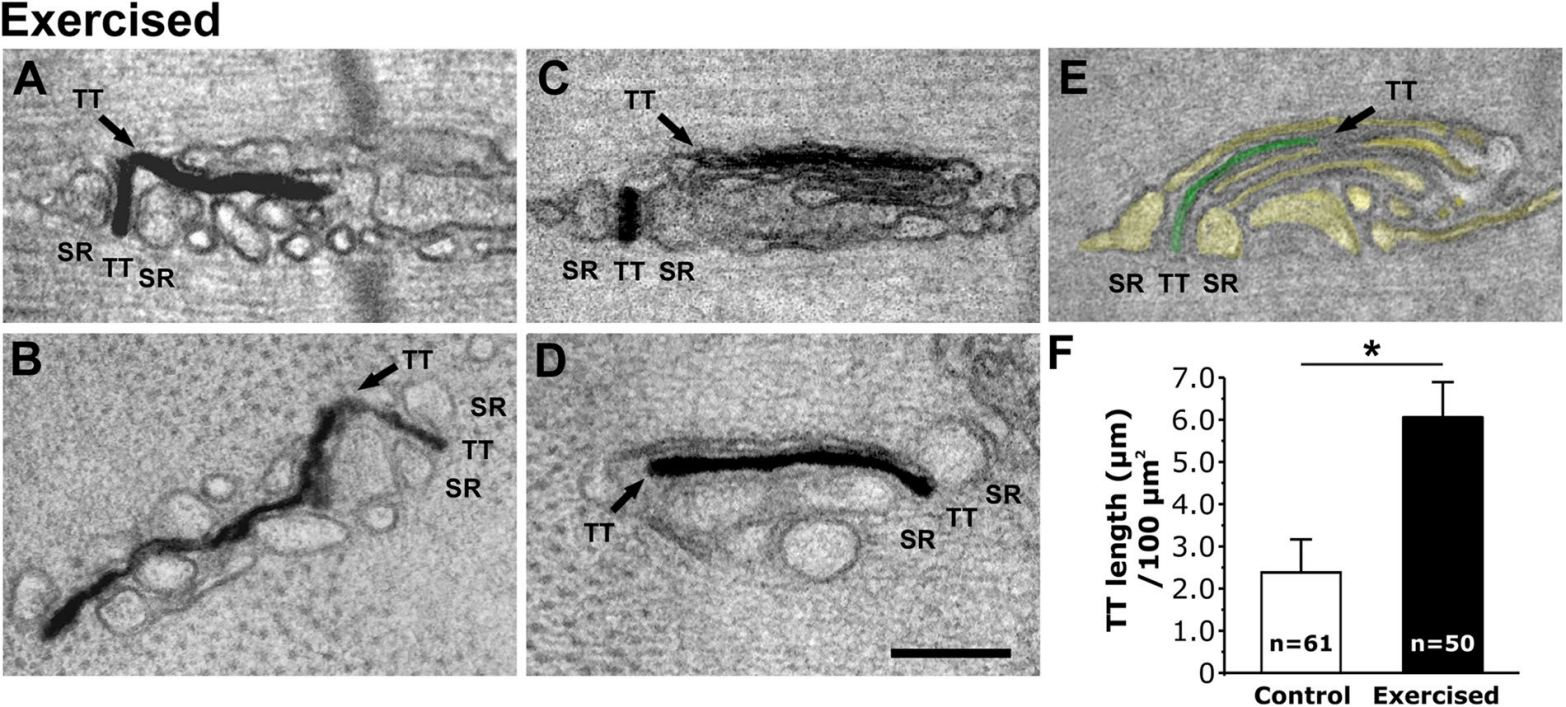
Following exercise, TT extensions at the I band are more frequent and become part of stacks of flat-cisternae. (**A**–**D**) Representative EM images (**A** and **C**, longitudinal; **B** and **D**, cross) showing TTs (stained black with ferrocyanide-precipitate) extending from the triad into the I band to make contact with SR vesicles (**A** and **B**) and stacks of flat-cisternae (**C** and **D**). (**E**) TT and SR (green and yellow, respectively) extending from a triad to form a lateral stack of flat-cisternae. (**F**) Quantitative analysis of the TT network extension at the I band measured in cross sections (see also Fig. S3C–F). *Sample size: control, 3 mice; exercised, 3 mice*. Data are shown as mean ± SEM; *p < 0.01. Numbers in bars (n) indicate the number of fibers analyzed. Scale bar: 0.1 μm.

We stained the TT network with ferrocyanide, a precipitate that enters the TT lumen from the extracellular space to enable direct visualization of TTs by EM (Fig. 2; and Fig. S3). While in control muscles, longitudinal extensions of the TT were occasionally observed in the I band (Fig. S3A,C and D), they were much more frequent following treadmill exercise (Fig. S3B,E and F). These TTs: a) extended longitudinally/obliquely into the I band region, while maintaining continuity with the transversal portion of the TT that constitutes the central element of the triad (Fig. 2A–D); and b) they were often seen associated with stacks of flat-cisternae (Fig. 2,D; inset in Fig. S3F). A direct continuity between a triadic TT profile and it’s I band extension associated within a stack of flat-cisternae is clearly visible in Fig. 2E. The increase of TT extensions into the I band following exercise was confirmed in EM cross sections, which enabled direct measurement of the total TT extension/100 μm^2^ (Fig. 2F).

### Exercise promotes STIM1-Orai1 co-localization at the I band

**Figure 3:**
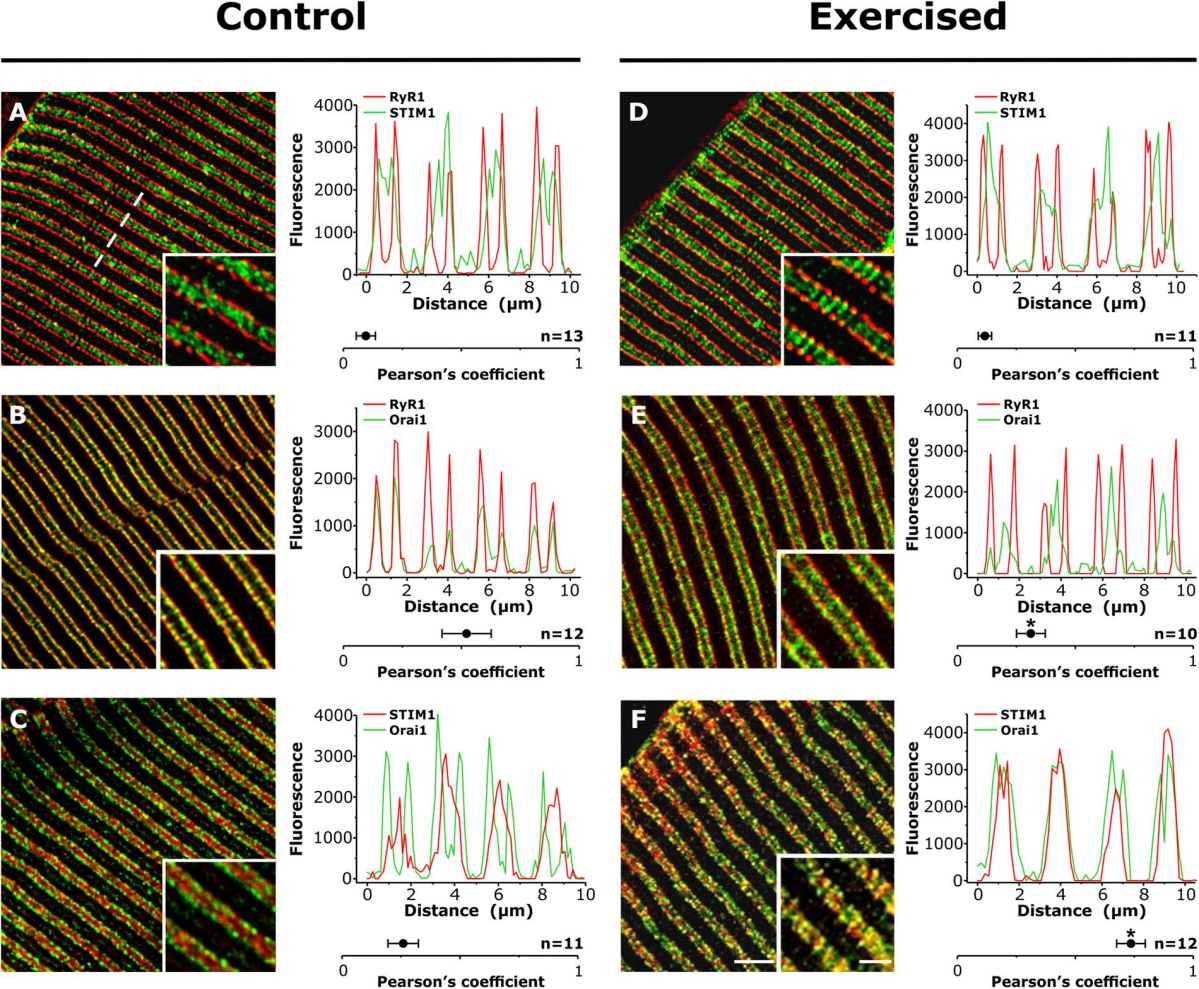
Co-localization between STIM1 and Orai1, low in control samples, increases significantly following exercise. Representative immunofluorescence images obtained from EDL fibers double-labeled for RyR1-STIM1 (**A** and **D**), RyR1-Orai1 (**B** and **E**), and STIM1-Orai1 (**C** and **F**). Each panel also contains a fluorescence intensity profile along 4 sarcomeres (see dashed line in **A**) and the Pearson’s correlation coefficient value, a measure of covariance of pixel intensities, given as the mean ± SEM; *p < 0.01, compared to fibers from control mice. Numbers in figure (n) indicate the number of images analyzed. Scale bar: 2.5 µm (insets: 1 µm).

To determine the subcellular localization of STIM1 and Orai1 under control conditions and following treadmill exercise, small bundles of EDL fibers were double-labeled in immunofluorescence experiments (Fig. 3) as follow: RyR1 vs. STIM1 (Fig. 3A,D), RyR1 vs. Orai1 (Fig. 3B,E), and STIM1 vs. Orai1 (Fig. 3C,F).

The staining of RyR1 was used as a known reference point marking the position of CRUs (i.e. triads): in adult fibers, triads were located at the A-I band junction on both sides of Z lines to form a double set of transverse cross striations, marked as RyR1-positive puncta (red signal in Fig. 3A,D). In resting muscle, STIM1 was localized throughout the entire I band region, with peaks of the green STIM1 signal of variable intensity (Fig. 3A). In muscles from exercised mice, STIM1 retained the same location at the I band, though exhibiting spots that appeared brighter and more organized as puncta than in control samples (compare insets in Fig. 3A,D). The low presence of yellow color, due to limited overlap between STIM1 and RyR1 fluorescence, and low Pearson’s correlation coefficient value are indicative of minimal STIM1-RyR1 co-localization under both control and exercised conditions (Fig. 3A,D). In contrast to STIM1, Orai1 labeling of control fibers resulted in significant co-localization with RyR1, as indicated by overlapping of peaks of RyR1 and Orai1 fluorescence, significant yellow signal in the merged image, and a relatively high Pearson’s correlation coefficient value (Fig. 3B). This labeling is consistent with a preferential localization of Orai1 in TTs at the triad in control samples. As a consequence of a high RyR1-Orai1 co-localization, the level of Orai1 co-localization with STIM1 at rest is low, as indicated by the relatively low Pearson’s correlation coefficient value (Fig. 3C).

While RyR1 and STIM1 positioning did not change following exercise (Fig. 3D–F), a fraction of the Orai1 signal shifted toward the I band in muscles from exercised mice (Fig. 3E and Fig. S3), resulting in a significant reduction of RyR1-Orai1 co-localization (compare Pearson’s correlation coefficient values in Fig. 3B,E; p < 0.01). As a consequence of this shift in Orai1 location, STIM1-Orai1 co-localization was significantly increased, as shown by superimposition of peaks of fluorescence (traces in Fig. 3F), increased yellow signal in the merged image (inset in Fig. 3F), and an increased Pearson’s correlation coefficient value (compare Pearson’s correlation coefficient values in Fig. 3C,F; p < 0.01). The increased presence of Orai1 at the I band is consistent with the elongation of Orai1-containing TT extensions toward the Z-line (see Fig. 2; and Fig. S3).

### Immunogold labeling confirms an increased presence of Orai1 in the I band following exercise

**Figure 4:**
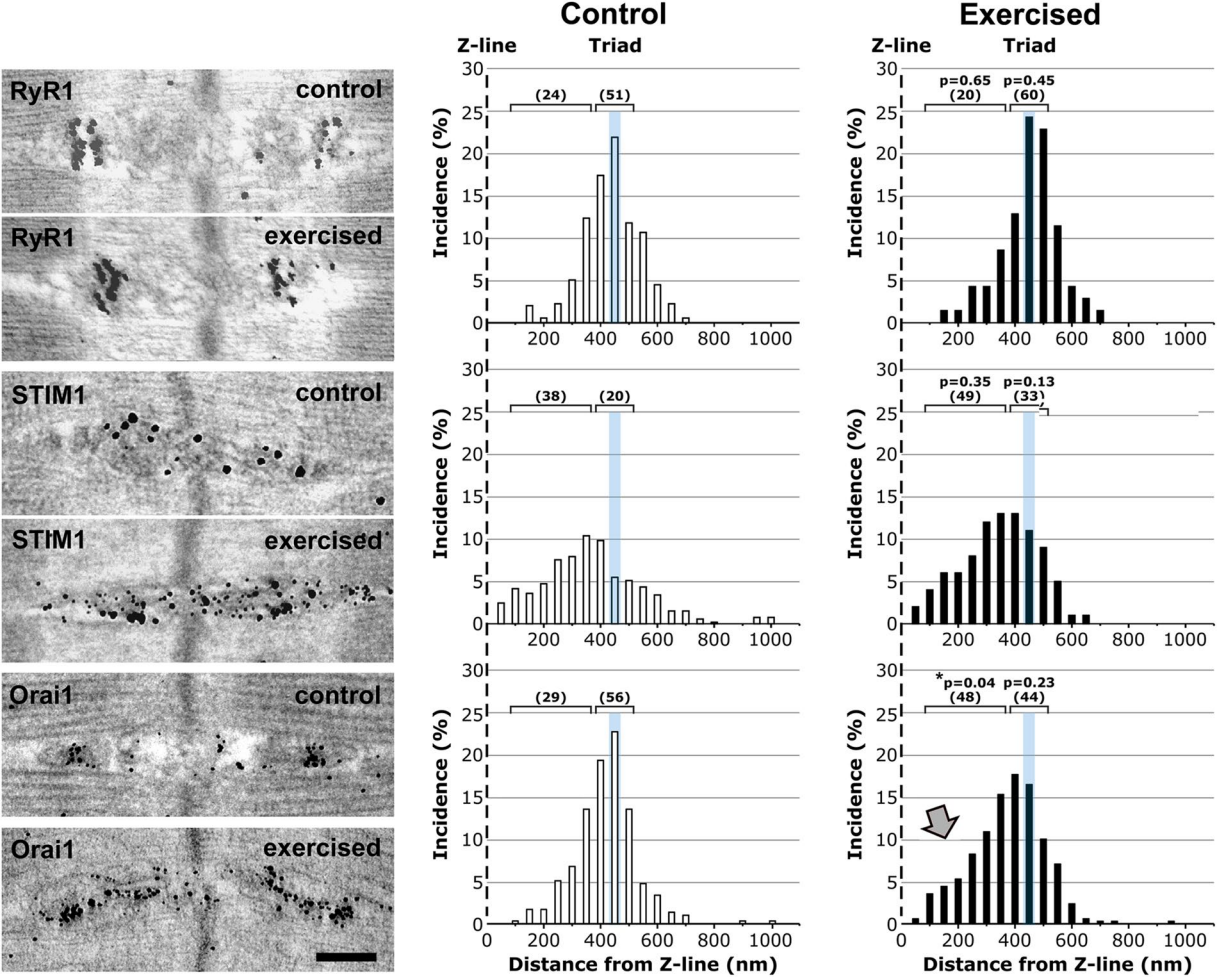
Immunogold labeling shows i) preferential I band localization of STIM1 and ii) increased presence of Orai1 at the I band following exercise. Left) Representative immunogold EM images showing the localization of RyR1 (top), STIM1 (center), and Orai1 (bottom) under control and exercised conditions. Right) Histograms of distances of immunogold particles from the Z line. The cyan line marks the position of the TT at the triad. Brackets with numeric values indicate the percentage of gold particles that fall within the triad-area vs. those that fall within the I-band area. Grey arrow points to the increased presence of Orai1 at the I-band following exercise. *Sample size: control, 2 mice/4–8 fibers analyzed; exercised, 3 mice/3–8 fibers analyzed*. *p < 0.05. Scale bar: 0.2 µm.

To better define the localization of STIM1 and Orai1 at the ultra-structural level, we performed immunogold (IG) labeling in muscles from control and exercised mice (Fig. 4). RyR1, STIM1, and Orai1 IG labeling in EM was then analyzed by generating histograms for the distance of each gold particle from the nearest Z-line (Fig. 4). As for immunofluorescence (Fig. 3), we used RyR1 antibodies to label the position of CRUs/triads, and marked their position with respect to the Z-line with a cyan bar in each of the histograms. The results in Fig. 4 indicate that:

- The position of both RyR1 and STIM1 did not change significantly following exercise: RyR1 remained at the triad and STIM1 remained preferentially present throughout the I band (Fig. 4-top and -center respectively; see also Fig. S4), even though some overlap between STIM1 and RyR1 was observed.
- Following exercise, the Orai1 signal shifted from the triad junction toward the I band (arrow in Fig. 4-bottom; see also Fig. S4), where remodeling of the sarcotubular system led to the formation of stacks of flat-cisternae (Figs 1 and S1). Note that these stacks contained electron-dense strands (Fig. S5A and B) that were specifically labeled by STIM1-immunogold particles (Fig. S5C and D).

The brackets with numeric-values above each histogram in Fig. 4 show the percentage of gold particles that fell within the *triad-area* vs. those that fell within the *I-band area* for each of the 3 proteins analyzed (RyR1, STIM1, and Orai1). Importantly, these analyses showed that, following exercise, while STIM1 levels increased both at the triad (from 20% to 33%) and I band (38% to 49%), Orai1 levels decreased in the triad (56% to 44%) and increased in the I band (between 100 and 350 nm from the Z-line; 29 to 48%). Additionally, non-linear curve fit analyses of the distribution of immunogold particle labeling for RyR1, STIM1, and Orai1 in EDL muscles from control (A) and exercised (B) mice confirmed a shift from the triad toward the I-band for only Orai1 (Fig. S4).

### EDL muscles from exercised mice exhibit enhanced resistance to repetitive high frequency fatigue when Ca^2+^ entry is not prevented

**Figure 5:**
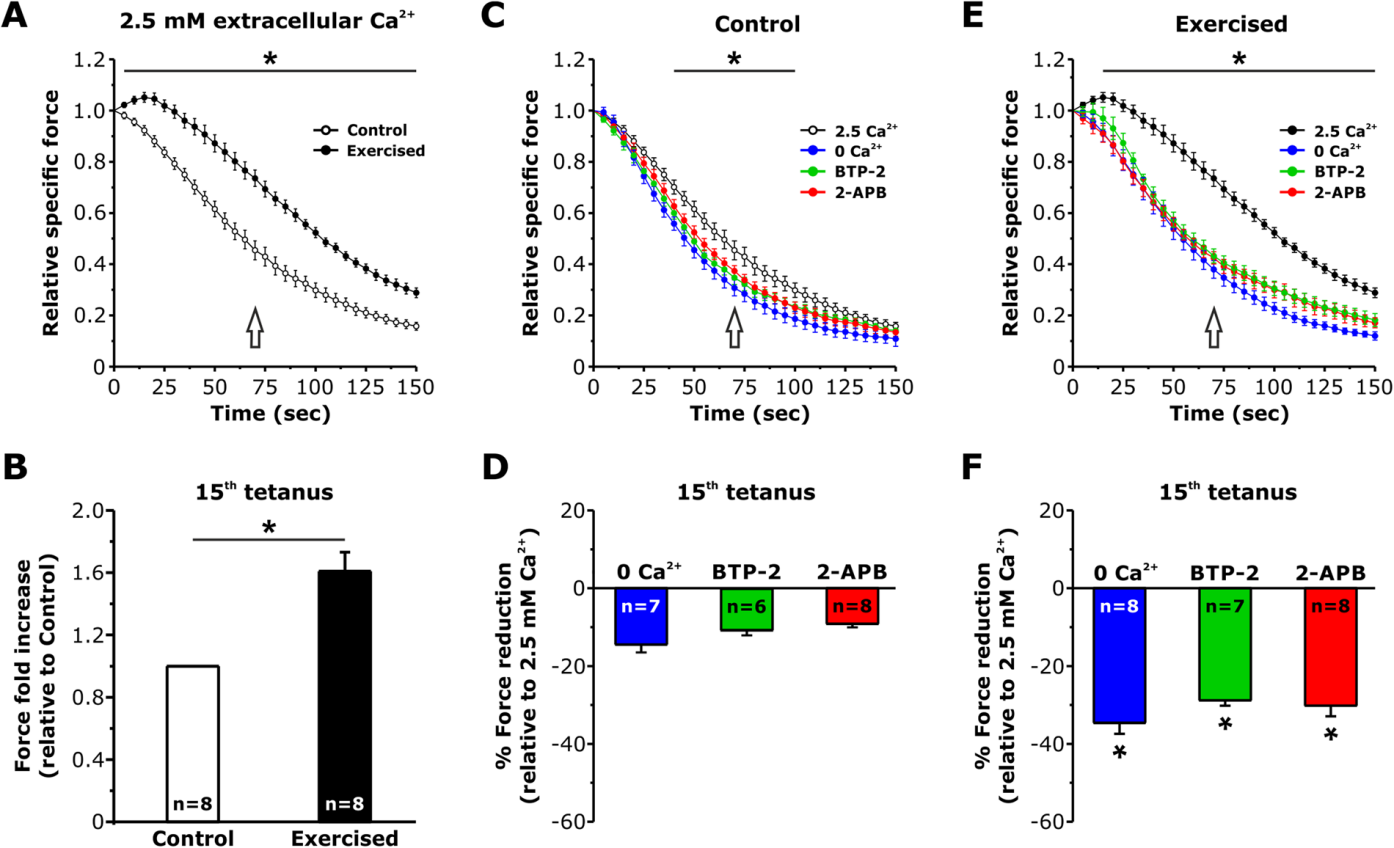
EDL muscles from exercised mice exhibit an enhanced resistant to fatigue that depends of Ca^2+^ entry. (**A**) Time course of average relative force decay during 30 consecutive frequency stimulus trains (1 s–60 Hz every 5 s), normalized to the 1^st^ stimulus train, in EDL muscles from control and exercised WT mice using a KH solution containing 2.5 mM Ca^2+^. (**B**) Bar plot showing the average fold increase in force produced by EDL muscles from exercised mice recorded during the 15^th^ stimulus train (arrow in **A**) normalized to that of control muscles. (**C** and **E**) Time courses of average relative force decay during repetitive stimulation of EDL muscles from control and exercised mice in the presence of 2.5 mM Ca^2+^, in Ca^2+^-free KH solution, or presence of either 10 µM BTP-2 or 100 µM 2-APB. (**D** and **F**) Bar plots summarizing average force reduction during the 15^th^ stimulus train in the presence of 2.5 mM Ca^2+^, in Ca^2+^-free KH solution, or presence of either 10 µM BTP-2 or 100 µM 2-APB. Data are shown as mean ± SEM; *p < 0.05. Number of experiments (n) reflect the number of EDL muscles analyzed for each condition.

We next compared the resistance to fatigue of EDL muscles excised from control and exercised mice. Before starting the fatigue protocol, muscles were first subjected to a standard force-frequency stimulation protocol using stimulus trains from 1 to 250 Hz. No difference was observed in either the peak specific force-frequency or normalized force-frequency curves for EDL muscles from control and exercised mice (Fig. S6A and B). However, EDL muscles from exercised mice exhibited an increased ability to maintain contractile force during a repetitive high frequency stimulation protocol (30 consecutive 1 s duration 60 Hz stimuli delivered every 5 seconds) compared to that of muscles from control mice (Fig. 5A,B; Fig. S7A and E). Specifically, the average fractional force during the 15^th^ stimulus train (Fig. 5A) was significantly greater in EDL muscles from exercised mice (0.74 ± 0.03) compared to that of EDL muscles from control mice (0.46 ± 0.03) (Fig. 5B). Similar results were observed when using a stimulation rate (100 Hz) at the peak of the force-frequency, though the magnitude of the increased ability to maintain contractile force was less than that observed at the lower frequency (Fig. S6C and D).

To assess the role of Ca^2+^ entry in the enhanced fatigue resistance of EDL muscles from exercised mice, parallel experiments were conducted using either a Ca^2+^-free KH solution in which Ca^2+^ was replaced with Mg^2+^ (Fig. 5C–F; and Fig. S1B and F) or a Ca^2+^-containing KH solution supplemented with either 10 μM BTP-2 (Fig. 5C–F; and Fig. S7C and G) or 100 μM 2-APB (Fig. 5C–F; and Fig. S7D and H), established inhibitors of SOCE^36–39^. These studies revealed that the enhanced fatigue resistance of EDL muscles from exercised mice was no longer significantly different from controls in either the absence of extracellular Ca^2+^ or presence of SOCE inhibitors. Specifically, the significant increase in force during the 15^th^ stimulus train observed in EDL muscles from exercised mice was no longer observed under conditions that prevented SOCE (Fig. 5C–F).

## Discussion

SOCE is a major pathway for Ca^2+^ influx from the extracellular space in skeletal muscle fibers. The importance of this process for muscle function is supported by prior studies demonstrating a potential role for SOCE in muscle fatigue^17,18,40^, ageing^19,20,41^, and myopathy^23,24,27–29^. In spite of these advances, the precise subcellular localization of proteins involved in SOCE (STIM1 and Orai1) in skeletal muscle fibers has not been unequivocally identified.

### Main findings

Our results indicate that: a) Exercise drives a remodeling of the existing sarcotubular system within the I band of the sarcomere that leads to the formation of stacks of membranes (Fig. 1 and Fig. S1) containing two distinct components: TT extensions into the I band (Fig. 2; and Fig. S3) and stacks of flat SR cisternae. b) The newly formed SR-TT junctions promote increased co-localization of STIM1 and Orai1 at the I band (Figs 3 and 4; and Fig. S4). While the frequency of new TT-SR junctions within the I band (i.e. distinct from triads) increases following treadmill exercise, these junctions are also present at a lower frequency and smaller in size in resting muscles (Fig. 1, Fig. S2, and Table 1), and thus, may contribute to sites of STIM1-Orai1 proximity under control conditions. c) EDL muscles isolated from exercised mice are more resistant to fatigue than EDL muscles from control mice (Fig. 5; Figs S6C and D; and S7). Moreover, this difference in fatigue susceptibility does not reflect a change in the force-frequency relationship, is observed at multiple frequencies of stimulation, and is markedly reduced by experimental interventions that reduce SOCE (e.g. removal of extracellular Ca^2+^ or addition of SOCE inhibitors^36,37^; Fig. 5 and Fig. S7).

### Main events leading to formation of new SR-TT junctions at the I band

**Figure 6:**
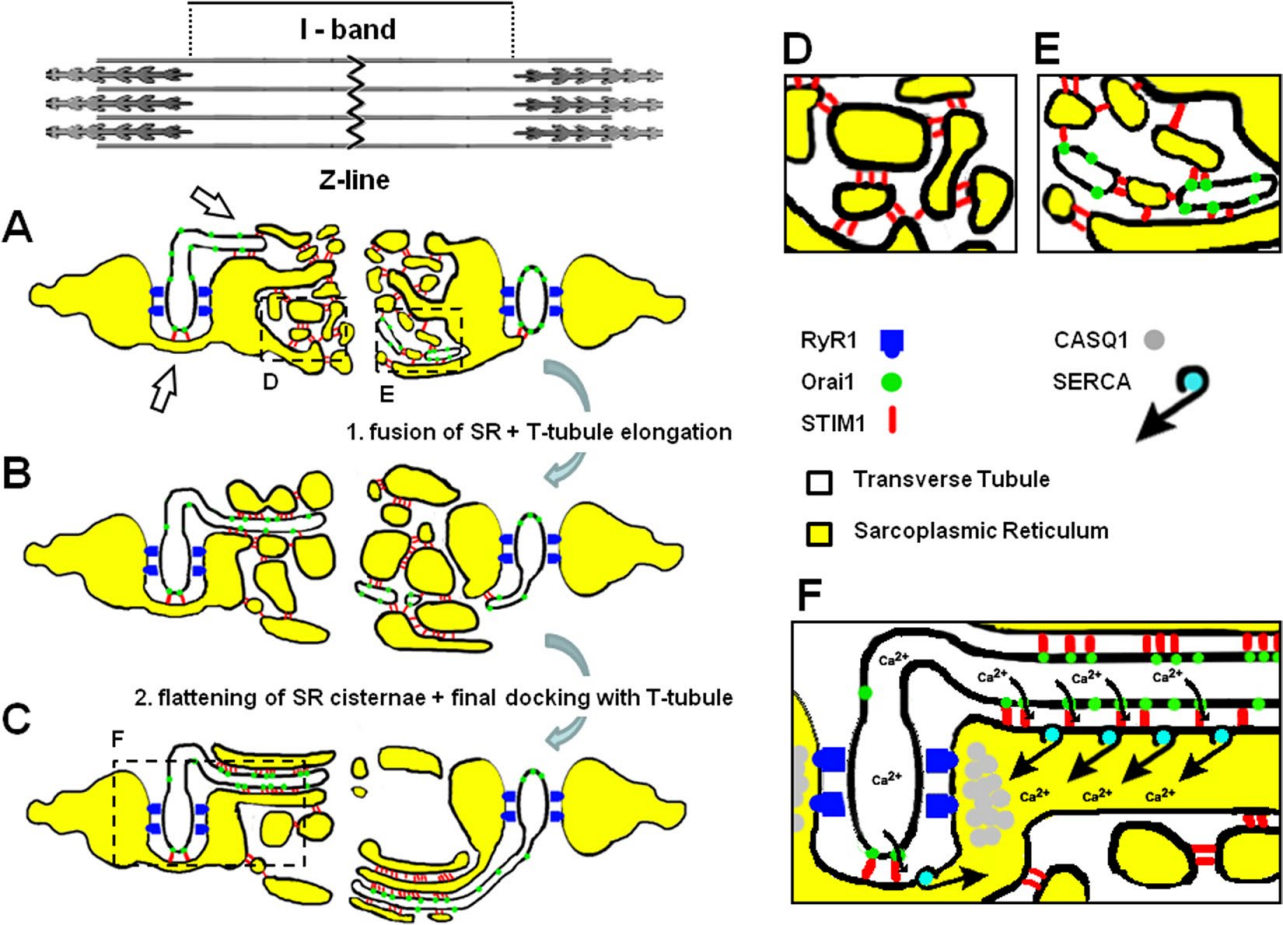
Model for exercise-dependent formation of Calcium Entry Units (CEUs). Main events leading to CEU formation: (**A**-to-**B**) fusion of free-SR into larger vesicles and elongation of TTs toward the I band; (**B**-to-**C**) flattening of SR membranes into parallel cisternae and final docking to elongated TTs. (**D** and **E**) Enlargements of dashed boxes in A: under control conditions, some membranes within the I band may (**E**) or may not (**D**) be of TT origin (and contain Orai1). (**F**) Enlargement of dashed box in C with modeling of the *Ca*^*2*+^*entry pathway* that would allow flow-back of ions to triads during exercise/fatigue. Empty arrows in A point to SR-TT contacts in proximity of triads, as imaged by EM in Fig. S8.

In non-muscle cells, store depletion triggers the formation of STIM1 aggregates within the ER, that then translocate to the surface membrane to trap Orai1 channels in STIM1-Orai1 *puncta*^8,11^. The findings of the present paper suggest that alternative mechanisms may coordinate STIM1-Orai1 assembly in skeletal muscle as, in contrast to non-excitable cells, the external membrane containing Orai1 proteins in skeletal muscle fibers (i.e. TTs) moves toward sites of STIM1 localization (within the I band). A model depicting the main events that occur during exercise-dependent formation of new SR-TT junctions within the I band is proposed in Fig. 6:

Figure 6A,B: fusion of free-SR (yellow) into larger tubules (Fig. 1H) and elongation of TTs (white) toward the I band (Fig. 2; and Fig. S3).

Figure 6B,C: flattening of SR membranes into parallel cisternae and docking to elongated TTs to form junctions that are connected to, but distinct from, the triad (Fig. 1; and Figs S1–S3).

In Fig. 6, while STIM1 (shown in red) is present throughout the I band both in control and exercised muscle (Fig. 6A), the latter is characterized by a remodeling of the SR (Fig. 6C). While primarily localized within TTs at the triad under control conditions (Fig. 6A), Orai1 (shown in green) translocates toward the Z-line following exercise, which likely results from an elongation of the TT into SR membrane stacks located deeper within the I band (Fig. 6C). The model presented in Fig. 6 also accounts for the following additional details:

STIM1 is localized both at the SR-TT and SR-SR interface. Indeed, similar electron-dense junctional strands were described previously in cells that overexpress only STIM1^35,42^, consistent with the idea that ER-ER stacks containing STIM1 molecules may form independently of the presence of Orai1^43^ and/or ER depletion (a more detailed Discussion of this issue in provided in Supplementary Information, Discussion: *STIM1 may be present at the SR-TT and SR-SR interface)*.

The existence of contacts between the SR and TT in close proximity to the triad could enable STIM1-Orai1 interactions under control conditions (empty arrows in Fig. 6A), a scenario supported by EM images presented in Fig. S8 (a detailed Discussion of this issue is provided below).

Some of the membranes within the I band observed under control conditions could be of TT origin and contain Orai1 proteins (Fig. 6A-right and enlargement in Fig. 6E), which would explain low-level Orai1 staining within the I band under resting conditions as observed in both immunofluorescence (Fig. 3B-inset) and immunogold (Fig. 4-bottom) experiments. However, the precise molecular mechanisms that control exercise-dependent remodeling of SR and TT membranes are currently unknown.

### Are the new SR-TT junctions involved in Ca^2+^ entry?

Using constitutive and inducible muscle-specific Orai1 knockout mice, Carrell *et al*.^18^ concluded that: a) acute Orai1-mediated Ca^2+^ influx plays a marginal role in limiting muscle fatigue in control (non-exercised) muscles; and b) Orai1 enhances muscle endurance primarily by promoting fatigue-resistant type I fiber content rather than via SOCE. Consistent with this conclusion, we found that co-localization of STIM1 and Orai1 is low in non-exercised muscles (see Figs 3 and 4; and Fig. S4) and contractile decline during repetitive high frequency stimulation of EDL muscle from sedentary mice is only minimally affected (Fig. 5C,D) by interventions that inhibit Ca^2+^ entry, e.g. 0 Ca^2+^ or the addition SOCE inhibitors BTP-2 and 2-APB^36,37^. Given the absence of an effect of Orai1 knockout in sedentary adult mice on EDL muscle fatigue during repetitive high frequency stimulation^18^, the small effect of Ca^2+^ entry on muscle fatigue in control muscles observed in Fig. 5C,D could be mediated by a compensatory increase in another store-operated Ca^2+^ channels (i.e. TRPC channels) in the knockout mice. In any event, we found that a single bout of treadmill exercise caused both the formation of new SR-TT contacts in the I band that significantly increases STIM1 and Orai1 co-localization and reduced fatigue during repetitive high frequency stimulation (Figs 3 and 4; for additional details see Supplementary Information Discussion: *Experimental evidence demonstrating the presence of both STIM1 and Orai1 in newly formed SR-TT junctions*). However, it is important to point out that, while increased co-localization in immunofluorescence and immunogold studies provide information about the proximity between the two proteins, this does not necessarily imply a direct interaction. The formation of SR-TT stacks following exercise results in EDL muscles that are more resistant to fatigue (Fig. 5F) than EDL muscles from control mice, a difference that is essentially abolished by experimental interventions that reduce Orai1-dependent SOCE (e.g. 0 Ca^2+^ or the addition SOCE inhibitors BTP-2 and 2-APB; Fig. S7). However, as these interventions (i.e. 0 Ca^2+^, BTP-2, and 2-APB) would inhibit multiple forms of Ca^2+^ entry, we cannot exclude the possibility that newly formed junctions also contain other types of Ca^2+^ entry channels other than Orai1 (i.e., TRPC^44^).

Nevertheless, we propose that: a) Orai1-mediated Ca^2+^ influx into the muscle following exercise limits fatigue and that this is achieved by forming new SR-TT junctions that promote STIM1-Orai1 coupling (Figs 1 and S1); and b) the new SR-TT junctions formed following exercise function as *Calcium Entry Units (CEUs)* during repetitive muscle activity by providing structural elements to coordinate STIM1-Orai1 coupling and a preferential pathway to refill the SR during prolonged muscle activity (Fig. 6F). As the SR at the I band is highly-enriched in sarco-endoplasmic reticulum Ca^2+^ATPases (SERCA)^45^, in principle the entry of extracellular Ca^2+^ could be efficiently re-sequestered by these pumps, while limiting diffusion of Ca^2+^ ions to the contractile elements (Fig. 6F). The exact contribution of SR-TT contacts in close proximity of triads (such as those shown in Fig. S8 and pointed by empty arrows in Fig. 6A) to Ca^2+^ entry at rest and during exercise remains to be determined. However, the combination of: i) a 5-fold increase in CEU frequency (Fig. 1H) relative to CRU frequency (resulting in a reduction in CRU/CEU ratio from 37:1 to 7:1; Table 1); and ii) increase CEU size with exercise (Fig. S2) suggest that the relative contribution of SOCE at the triad would decrease compared to that occurring via CEUs following exercise.

### Relationship of present findings to prior studies in muscle

Stacks of flat-cisternae virtually identical to those displayed in Fig. 1 and Fig. S1 were previously observed in muscle from transgenic mice with junctophilin knockdown (referred to as modified-triads^46^), in CASQ1 and CASQ2 knockout^47,48^ mice, and in triadin-junctin double knockout mice (described as SR-stacks^49^). Here we show that these structures also contain TT membranes, the molecular components for SOCE (STIM1 and Orai1, but not RyR1), exhibit a significantly smaller junctional gap than that of the triad (Fig. 1G), increase in number and size following exercise, and thus, constitute a newly-identified structural unit or organelle in skeletal muscle.

Prior studies have demonstrated that SOCE in skeletal muscle involves STIM1-Orai1 coupling^15,17,50^ and can be rapidly activated upon store depletion^13,14,23,32^. As our results indicate that formation of CEUs during exercise requires a relatively long time to assemble, it is unlikely that newly formed CEUs underlies rapid SOCE activation^13,32^. Two observations of the present study help to reconcile this apparent discrepancy: 1) preformed CEUs are present in muscle fibers under control (i.e. non-exercised) conditions, although they occur at a lower frequency and are smaller in size (Fig. 1; and Fig. S2); and 2) small electron-dense strands connecting terminal SR membranes with the lateral side of TTs are visible in close proximity of some triads (see Fig. S8; empty arrows in Fig. 6A). In fact, since the CRUs vastly out-number CEUs (37:1) under non-exercised control conditions, even a small density of STIM1-Orai1 co-localization within these lateral triad contacts (see Fig. S8 and Fig. 6A, arrows) could significantly contribute to rapid SOCE activation^13,14^ even in the absence of exercise-dependent membrane rearrangements.

### Final remarks

Impaired muscle function and/or increased fatigability contributes to a reduction in the quality of life of many individuals. In this context, activity-dependent formation of junctions containing STIM1 and Orai1 proteins used to facilitate Ca^2+^ entry and SR Ca^2+^ store replenishment may represent one beneficial component of muscle adaptation during fatigue. As altered SOCE^41^ and mutations in STIM1 and Orai1 contribute to muscle dysfunction in ageing and myopathies^18,19,22,23,26,41^, future investigations of CEU formation and function may lead to an improved the understanding of these pathophysiological conditions.

## Materials and Methods

All experiments were conducted in male mice housed in microisolator cages under identical conditions. Mice were either sacrificed before *(control)* or after being subjected to a single bout of treadmill running *(exercised)*.

### Animals

C57bl/6 mice were housed in microisolator cages at 20 °C in a 12-h light/dark cycle and provided free access to standard chow and water. Four-month old male C57bl/6 mice were randomly assigned to two experimental groups: control and exercised (mice exposed to a single bout of exercise protocol; see below). All experiments were conducted according to the Directive of the European Union 2010/63/UE and were approved by the Animal Ethical Committees of the University of Chieti. All surgeries were made to minimize animal suffering, i.e., animals were sacrificed by cervical dislocation as approved by the local University Committee on Animal Resources (15/2011/CEISA/COM).

### Treadmill exercise protocol

Experiments were performed at room temperature using a running treadmill (Columbus Instruments). A first step of warm-up at low speed (10 min at 5 meters/min) was used to familiarize the mice with the apparatus and task. The experimental exercise protocol started immediately after the warm-up session and was designed as follows: at the beginning of the protocol, the speed was set to 10 m/min for 25 minutes, then to 15 m/min for 20 minutes, then to 20 m/min for 15 minutes, and finally the speed was increased for 1 m/min every 1 minute until the final speed of 25 m/min was reached (and kept for maximum 1 minute). The protocol was stopped when mice reached exhaustion, evaluated as the inability of the animal to maintain running speed and contact with the treadmill. Variability between different mice was limited: some mice stopped just before or during the last 5 minutes of the protocol (when the speed was increased each minute by 1 m/min), while other mice were able to continue running until the end of the protocol.

### Electron Microscopy (EM)

EDL muscles were dissected from sacrificed animals, pinned on a Sylgard dish, fixed at room temperature with 3.5% glutaraldehyde in 0.1 M sodium cacodylate (NaCaCo) buffer (pH 7.2), and then stored in the fixative at 4 °C. Small bundles of fixed muscle were then post-fixed, embedded, stained en-block, and sectioned for EM, as described previously^51^. For TT staining, specimens were post-fixed in a mixture of 2% OsO_4_ and 0.8% K_3_Fe(CN)_6_ for 1–2 h followed by a rinse with 0.1 M NaCaCo buffer with 75 mM CaCl_2_. Ultrathin sections (~50 nm) were cut using a Leica Ultracut R microtome (Leica Microsystem) with a Diatome diamond knife (Diatome Ltd.) and double-stained with uranyl acetate and lead citrate. Sections were viewed in a FP 505 Morgagni Series 268D electron microscope (FEI Company), equipped with Megaview III digital camera and Soft Imaging Systemat 60 kV or 100 kV (for TT staining preparations).

### Immunogold labeling for EM

EDL muscles were fixed for 20 min at room temperature in a fixative mix containing 2% paraformaldehyde and 0.5% glutaraldehyde (in PBS buffer). Small bundles of fixed fibers were blocked for 1 hour in PBS containing 1% bovine serum albumin (BSA), 10% goat serum, and permeabilized with 0.5% TritonX-100. After incubation with primary antibodies, secondary antibodies conjugated with Nanogold® particles (either goat anti-mouse or goat anti-rabbit; Nanoprobes) were applied for 2 h at 4 °C (1:100). Samples were then post-fixed in 1% glutaraldehyde PBS buffer at room temperature and incubated with reagents to enhance the signal (Goldenhance™ EM Formulation, Nanoprobes) for 5 minutes. Fiber bundles were finally post-fixed, embedded, and sectioned with standard EM protocols. Primary antibodies used: mouse monoclonal anti-RyR1/RyR3 34 C antibody, (1:30, Developmental Studies Hybridoma Bank); rabbit polyclonal anti-STIM1, (1:100, Sigma Aldrich); rabbit polyclonal anti-Orai1, (1:20, Thermo Scientific).

### Quantitative analysis of EM and immunogold preparations

- The percentage of fibers exhibiting *stacks* of flat-long cisternae/tubes and the number of stacks per 100 µm^2^ area/section (data in Fig. 1E,F) were determined from electron micrographs of non-overlapping regions randomly collected from transverse sections. In each specimen, 9–21 cross-sectional fibers were analyzed and in each fiber 5 micrographs were taken at 28,000x magnification.
- The junctional gap in CRUs (or triads), between SR vesicles (in control) and in stacks of flat-cisternae in electron micrographs were taken at 44,000x or 56,000x magnification (data in Fig. 1G).
- The average size of SR vesicles in proximity of *stacks* (data in Fig. 1H) was measured in transverse sections from electron micrographs at 44,000x magnification.
- The extension of non-triadic TT network within at the I band (data in Fig. 2F) was measured in electron micrographs at 28,000x magnification of non-overlapping regions randomly collected from transverse sections of samples stained with ferrocyanide (see also Fig. S3). In each specimen, 9–21 cross-sectioned fibers were analyzed and in each fiber 5 micrographs were taken.
- The average length of the membrane-membrane contacts in the stacks of flat-cisternae was measured in micrographs taken at 56,000x magnification as shown in Fig. S2C (labeled by cyan dots).
- The number of membrane elements contained in the stacks of flat-cisternae (data in Fig. S2D) was obtained in micrographs taken at 28.000x and 44,000x magnification by counting single elements clearly delimited by membrane phospholipids.
- The number of CRUs and CEUs in 100 µm^2^ of EM section (data in Table 1) was determined from electron micrographs of non-overlapping regions randomly collected from longitudinal sections. In each specimen, 7 fibers were analyzed, and in each fiber, 5–10 micrographs were taken at 18,000x magnification.
- The distance of gold particles from the nearest Z line in longitudinal sections was measured in immunogold labeled EM sections (data in Fig. 4; and Fig. S4). Quantitative data from electron micrographs in sections b), c), d), e), and h) (above) were obtained using the Analy-SIS software of the EM digital camera (Olympus Soft Imaging Solutions).

### Immunofluorescence labeling and confocal microscopy (CM)

EDL muscles were fixed and processed for immunofluorescence as previously described^51^. Primary antibodies used: a) mouse monoclonal anti-RyR1/RyR3 (34 C antibody, 1:30, Developmental Studies Hybridoma Bank); b) rabbit polyclonal anti-STIM1 (1:100, Sigma Aldrich); c) mouse monoclonal anti-STIM1 (1:50, BD Biosciences); or d) rabbit polyclonal anti-Orai1, (1:20, Thermo Scientific). Secondary antibodies used: a) Cy5-labeled goat anti-mouse IgG (1:50); or b) Cy3-labeled goat anti-rabbit (1:200). Confocal images were acquired using a Zeiss LSM510 META system equipped with a Zeiss Axiovert 200 inverted microscope and a Plan Neofluar oil-immersion objective (100X/1.3 NA). Fluorescence image profiles were obtained from LSM 3.0 image analysis software by Zeiss. Co-localization was determined by Imaris 7.2.3 software (Bitplane) and quantified by an evaluation of the Pearson’s correlation coefficient values.

### *Ex-vivo* fatigue protocol

Intact EDL muscles were removed from hind limbs of control and exercised mice and placed in a dish with a Krebs-Henseleit (KH) solution containing: 118 mM NaCl; 5 mM KCl; 2.5 mM CaCl_2_; 1 mM KH_2_PO_4_; 1 mM MgSO_4_; 25 mM NaHCO_3_; and 11 mM Glucose; pH 7.4. Muscles were pinned, tied with fine silk sutures at each end, and mounted vertically between two platinum electrodes immersed in an organ chamber filled with KH solution^52^ and attached to a servo motor and force transducer (1200 A, Aurora Scientific). Before starting the experimental protocol, stimulation level and optimal muscle length (*L*_0_) were determined using a series of 80 Hz stimulation trains in order to stretch the muscle to the length that generated maximal force (*F*_0_). Twitch and tetanic contractile properties, as well as force-frequency parameters, were then measured. Following these baseline measurements, EDL muscles were then subjected to a repetitive stimulation fatigue protocol consisting of 30 consecutive stimulus trains at 60 Hz of frequency (each pulse having a duration of 1 s) applied every 5 s while being continuously perfused with KH solution. To assess the relative contribution of extracellular Ca^2+^ entry, some experiments were conducted either in the absence of external Ca^2+^ (equimolar substitution with Mg^2+^) or in presence of 10 µM BTP-2 or 100 µM 2-APB, two established inhibitors of SOCE^36–39^. In a separate set of experiments, EDL muscles from control and exercised mice perfused with control KH solution were subjected to a similar repetitive stimulation protocol, but using a simulation frequency (100 Hz) at the peak of the force frequency curve (i.e. 30 consecutive 1s-stimulus trains at 100 Hz of frequency applied every 5 s). Specific force (mN/mm^2^) was calculated by normalizing the absolute force (mN) to the physiological cross sectional area (in mm^2^) obtained as follows: wet weight (mg)/*(L*_0_ (mm) × 1.06 (mg/mm^3^) × 0.44). All experiments were carried out at room temperature.

### Statistical analyses

Statistical significance in all experiments was evaluated using a two-tailed unpaired Student’s t test for the 95% confidence intervals, with the exception of statistical significance in Figs 4 and S2F (determined using a two-tailed Fisher’s exact test) and the *ex vivo* muscle fatigue results of Figs 5, S6 and S7 (evaluated using a one-way ANOVA followed by *post-hoc* Tuckey test for multiple comparisons). All data were presented as mean ± SEM.

## Electronic supplementary material

Supplementary Information

## References

[1] Putney JW (1986). A model for receptor-regulated calcium entry. Cell Calcium.

[2] Parekh AB, Penner R (1997). Store depletion and calcium influx. Physiol Rev.

[3] Roos J (2005). STIM1, an essential and conserved component of store-operated Ca^2+^ channel function. J Cell Biol.

[4] Liou J (2005). STIM is a Ca^2+^ sensor essential for Ca^2+^ store-depletion-triggered Ca^2+^ influx. Curr Biol.

[5] Zhang SL (2005). STIM1 is a Ca^2+^ sensor that activates CRAC channels and migrates from the Ca^2+^ store to the plasma membrane. Nature.

[6] Feske S (2006). A mutation in Orai1 causes immune deficiency by abrogating CRAC channel function. Nature.

[7] Vig M (2006). CRACM1 is a plasma membrane protein essential for store-operated Ca^2+^ entry. Science.

[8] Wu MM, Buchanan J, Luik RM, Lewis RS (2006). Ca2+ store depletion causes STIM1 to accumulate in ER regions closely associated with the plasma membrane. J Cell Biol.

[9] Luik RM, Wang B, Prakriya M, Wu MM, Lewis RS (2008). Oligomerization of STIM1 couples ER calcium depletion to CRAC channel activation. Nature.

[10] Liou J, Fivaz M, Inoue T, Meyer T (2007). Live-cell imaging reveals sequential oligomerization and local plasma membrane targeting of stromal interaction molecule 1 after Ca^2+^ store depletion. Proc Natl Acad Sci USA.

[11] Lewis RS (2007). The molecular choreography of a store-operated calcium channel. Nature.

[12] Kurebayashi N, Ogawa Y (2001). Depletion of Ca^2+^ in the sarcoplasmic reticulum stimulates Ca^2+^ entry into mouse skeletal muscle fibres. J Physiol.

[13] Launikonis BS, Rios E (2007). Store-operated Ca^2+^ entry during intracellular Ca^2+^ release in mammalian skeletal muscle. J Physiol.

[14] Launikonis BS, Barnes M, Stephenson DG (2003). Identification of the coupling between skeletal muscle store-operated Ca^2+^ entry and the inositol trisphosphate receptor. Proc Natl Acad Sci USA.

[15] Lyfenko AD, Dirksen RT (2008). Differential dependence of store-operated and excitation-coupled Ca^2+^ entry in skeletal muscle on STIM1 and Orai1. J Physiol.

[16] Dirksen RT (2009). Checking your SOCCs and feet: the molecular mechanisms of Ca^2+^ entry in skeletal muscle. J Physiol.

[17] Wei-Lapierre L, Carrell EM, Boncompagni S, Protasi F, Dirksen RT (2013). Orai1-dependent calcium entry promotes skeletal muscle growth and limits fatigue. Nat Commun.

[18] Carrell Ellie M., Coppola Aundrea R., McBride Helen J., Dirksen Robert T. (2016). Orai1 enhances muscle endurance by promoting fatigue-resistant type I fiber content but not through acute store-operated Ca2+ entry. The FASEB Journal.

[19] Zhao X (2008). Compromised store-operated Ca^2+^ entry in aged skeletal muscle. Aging Cell.

[20] Thornton AM (2011). Store-operated Ca^2+^ entry (SOCE) contributes to normal skeletal muscle contractility in young but not in aged skeletal muscle. Aging (Albany NY).

[21] Payne AM, Jimenez-Moreno R, Wang ZM, Messi ML, Delbono O (2009). Role of Ca^2+^, membrane excitability, and Ca^2+^ stores in failing muscle contraction with aging. Exp Gerontol.

[22] Edwards JN, Blackmore DG, Gilbert DF, Murphy RM, Launikonis BS (2011). Store-operated calcium entry remains fully functional in aged mouse skeletal muscle despite a decline in STIM1 protein expression. Aging Cell.

[23] Zhao Xiaoli, Moloughney Joseph G., Zhang Sai, Komazaki Shinji, Weisleder Noah (2012). Orai1 Mediates Exacerbated Ca2+ Entry in Dystrophic Skeletal Muscle. PLoS ONE.

[24] Goonasekera SA (2014). Enhanced Ca^2+^ influx from STIM1-Orai1 induces muscle pathology in mouse models of muscular dystrophy. Hum Mol Genet.

[25] Onopiuk M (2015). Store-operated calcium entry contributes to abnormal Ca^2+^ signalling in dystrophic mdx mouse myoblasts. Arch Biochem Biophys.

[26] Pan Z, Brotto M, Ma J (2014). Store-operated Ca^2+^ entry in muscle physiology and diseases. BMB Rep.

[27] Bohm J (2013). Constitutive activation of the calcium sensor STIM1 causes tubular-aggregate myopathy. Am J Hum Genet.

[28] Nesin V (2014). Activating mutations in STIM1 and ORAI1 cause overlapping syndromes of tubular myopathy and congenital miosis. Proc Natl Acad Sci USA.

[29] Endo Y (2015). Dominant mutations in ORAI1 cause tubular aggregate myopathy with hypocalcemia via constitutive activation of store-operated Ca^2+^ channels. Hum Mol Genet.

[30] Okuma Hidehiko, Saito Fumiaki, Mitsui Jun, Hara Yuji, Hatanaka Yuki, Ikeda Miki, Shimizu Teruo, Matsumura Kiichiro, Shimizu Jun, Tsuji Shoji, Sonoo Masahiro (2016). Tubular aggregate myopathy caused by a novel mutation in the cytoplasmic domain ofSTIM1. Neurology Genetics.

[31] Garibaldi M., Fattori F., Riva B., Labasse C., Brochier G., Ottaviani P., Sacconi S., Vizzaccaro E., Laschena F., Romero N.B., Genazzani A., Bertini E., Antonini G. (2016). A novel gain-of-function mutation inORAI1causes late-onset tubular aggregate myopathy and congenital miosis. Clinical Genetics.

[32] Edwards JN (2010). Ultra-rapid activation and deactivation of store-operated Ca(2+) entry in skeletal muscle. Cell Calcium.

[33] Schneider MF (1994). Control of calcium release in functioning skeletal muscle fibers. Annu Rev Physiol.

[34] Franzini-Armstrong C, Protasi F, Ramesh V (1999). Shape, size, and distribution of Ca^2+^ release units and couplons in skeletal and cardiac muscles. Biophysical Journal.

[35] Orci L (2009). From the Cover: STIM1-induced precortical and cortical subdomains of the endoplasmic reticulum. Proc Natl Acad Sci USA.

[36] Zitt C (2004). Potent inhibition of Ca2 + release-activated Ca^2+^ channels and T-lymphocyte activation by the pyrazole derivative BTP2. J Biol Chem.

[37] Bootman MD (2002). 2-aminoethoxydiphenyl borate (2-APB) is a reliable blocker of store-operated Ca^2+^ entry but an inconsistent inhibitor of InsP3-induced Ca^2+^ release. FASEB J.

[38] Hendron E (2014). Potent functional uncoupling between STIM1 and Orai1 by dimeric 2-aminodiphenyl borinate analogs. Cell Calcium.

[39] Ohga K, Takezawa R, Arakida Y, Shimizu Y, Ishikawa J (2008). Characterization of YM-58483/BTP2, a novel store-operated Ca^2+^ entry blocker, on T cell-mediated immune responses *in vivo*. Int Immunopharmacol.

[40] Zhao X (2005). Enhanced resistance to fatigue and altered calcium handling properties of sarcalumenin knockout mice. Physiol Genomics.

[41] Brotto M (2011). Aging, sarcopenia and store-operated calcium entry: a common link?. Cell Cycle.

[42] Perni S, Dynes JL, Yeromin AV, Cahalan MD, Franzini-Armstrong C (2015). Nanoscale patterning of STIM1 and Orai1 during store-operated Ca^2+^ entry. Proc Natl Acad Sci USA.

[43] Park CY (2009). STIM1 clusters and activates CRAC channels via direct binding of a cytosolic domain to Orai1. Cell.

[44] He LP, Hewavitharana T, Soboloff J, Spassova MA, Gill DL (2005). A functional link between store-operated and TRPC channels revealed by the 3,5-bis(trifluoromethyl)pyrazole derivative, BTP2. J Biol Chem.

[45] Franzini-Armstrong C, Ferguson DG (1985). Density and disposition of Ca2 + -ATPase in sarcoplasmic reticulum membrane as determined by shadowing techniques. Biophys J.

[46] Ko JK (2011). A versatile single-plasmid system for tissue-specific and inducible control of gene expression in transgenic mice. FASEB J.

[47] Boncompagni S, Protasi F, Franzini-Armstrong C (2012). Sequential stages in the age-dependent gradual formation and accumulation of tubular aggregates in fast twitch muscle fibers: SERCA and calsequestrin involvement. Age (Dordr).

[48] Valle G (2016). Characterization of fast-twitch and slow-twitch skeletal muscles of calsequestrin 2 (CASQ2)-knock out mice: unexpected adaptive changes of fast-twitch muscles only. J Muscle Res cell Motil.

[49] Boncompagni Simona, Thomas Monique, Lopez Jose R., Allen Paul D., Yuan Qunying, Kranias Evangelia G., Franzini-Armstrong Clara, Perez Claudio F. (2012). Triadin/Junctin Double Null Mouse Reveals a Differential Role for Triadin and Junctin in Anchoring CASQ to the jSR and Regulating Ca2+ Homeostasis. PLoS ONE.

[50] Stiber J (2008). STIM1 signalling controls store-operated calcium entry required for development and contractile function in skeletal muscle. Nat Cell Biol.

[51] Pietrangelo L (2015). Age-dependent uncoupling of mitochondria from Ca^2+^ release units in skeletal muscle. Oncotarget.

[52] Michelucci A (2015). Antioxidants protect calsequestrin-1 knockout mice from halothane- and heat-induced sudden death. Anesthesiology.

