# Addendum: Exercise-dependent formation of new junctions that promote STIM1-Orai1 assembly in skeletal muscle

**DOI:** 10.1038/s41598-018-33063-0

**Published:** 2018-11-27

**Authors:** Simona Boncompagni, Antonio Michelucci, Laura Pietrangelo, Robert T. Dirksen, Feliciano Protasi

**Affiliations:** 1 CeSI-Met - Center for Research on Ageing and Translational Medicine, University G. d’Annunzio, Chieti, I-66100 Italy; 2 DNICS - Department of Neuroscience, Imaging and Clinical Sciences, University G. d’Annunzio, Chieti, I-66100 Italy; 3 Department of Pharmacology and Physiology, University of Rochester Medical Center, Rochester, NY 14642 USA; 4 DMSI - Department of Medicine and Aging Science, University G. d’Annunzio, Chieti, I-66100 Italy

Addendum to: *Scientific Reports* 10.1038/s41598-017-14134-0, published online 27 October 2017

In this Article, we used an antibody from Thermo Scientific (PA5-26378) in immunofluorescence (IF) and immuno-gold (IG) for electron microscopy (EM) studies to determine the subcellular localization of Orai1 channels in skeletal muscle fibers of *extensor digitorum longus* (EDL) muscle from mice at rest and following acute treadmill exercise. Our conclusions regarding the unexpected translocation of Orai1 toward STIM1 in the I band depend on the specificity of the Orai1 antibody used in our IF and IG studies. Given legitimate concerns raised regarding the relative specificity of commercially-available Orai1 antibodies (personal communication: Dr Robyn Murphy), we conducted parallel IF studies using the same Thermo Scientific antibody (PA5-26378) in EDL muscle fibers from control and tamoxifen-inducible, muscle-specific Orai1 knockout (iOrai1 KO) mice. The results of these studies, shown in new Fig. 1 included below, validate that the Thermo Scientific Orai1 antibody (PA-26378) used in the Article is highly-specific for Orai1 in IF experiments in intact skeletal muscle fibers.

**Figure 1:**
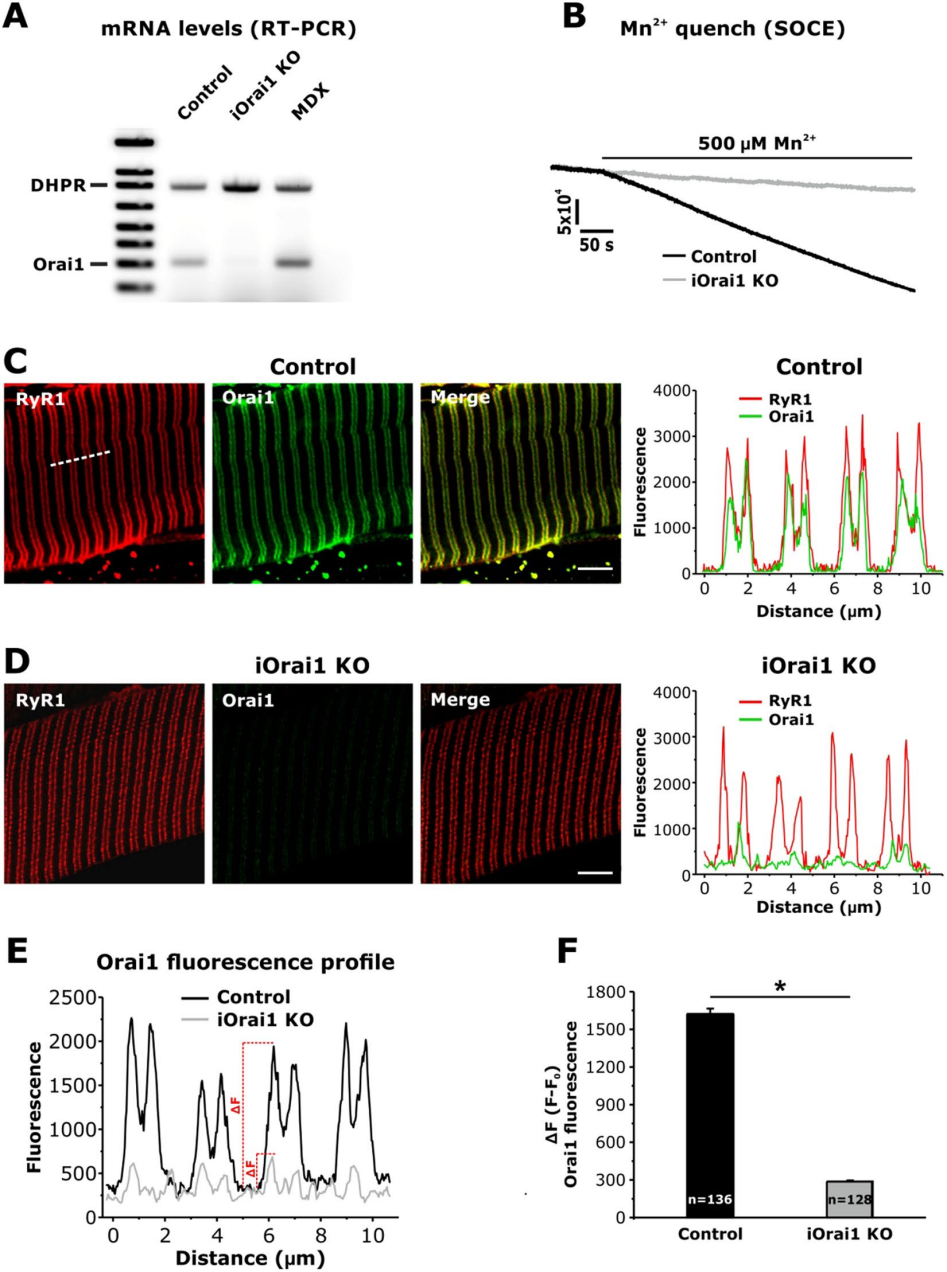
Validation of the specificity of the Thermo Scientific Orai1 antibody (PA-26378) for IF studies in murine skeletal muscle fibers. (**A**) Representative RT-PCR gel showing Orai1 mRNA levels in TA muscle homogenates from control, iOrai1 KO and mdx mice (MDX). DHPR mRNA was used as a loading control. (**B**) Representative superimposed traces of Mn^2+^ quench of fura-2 fluorescence emission recorded in single thapsigargin-treated FDB fibers obtained from control (black) and iOrai1 KO (grey) mice. (**C** and **D**) Representative raw and merged immunofluorescence images obtained from control (**C)** and iOrai1 KO (**D**) EDL fibers double-labeled for RyR1 (34C, red signal, left panels) and Orai1 (PA-26378, green signal, middle panels). Each set of panels includes a fluorescence intensity profile (right) across 4 consecutive sarcomeres (see dashed line in C, left panel). (**E**) Representative fluorescence intensity profile along 4 sarcomeres for Orai1 fluorescence signals in EDL fibers from control (black) and iOrai1 KO (grey) mice. (**F**) Quantitative analysis (ΔF = F − F0) of the Orai1 fluorescence signal calculated as the difference between the fluorescence signal (ΔF) at the triad (**F**) and the fluorescence signal localized at the A band (F0). Numbers in bars (n) reflect the total number of Orai1 fluorescence peaks analyzed across all fibers; 14 and 12 fibers were analyzed for control and iOrai1 KO mice, respectively; *p < 0.05 (unpaired Student’s t-test). Data are shown as mean ± SEM.

We used 4 month old male skeletal muscle-specific, tamoxifen-inducible Orai1 KO mice (*Orai1*^*fl/fl:HSA-MCM*^) that were fed either normal (control) or tamoxifen-infused chow to induce knock-out of the Orai1 gene (iOrai1 KO) for 4 weeks prior to isolating muscles and then quantifying: (1) Orai1 transcript levels in *tibialis anterior* (TA) muscles (Fig. 1A); (2) maximum rate of Mn^2+^ quench following thapsigargin-induced store depletion in single, acutely dissociated *flexor digitorum brevis* (FDB) fibers (Fig. 1B); and (3) IF localization of Orai1 in isolated EDLmuscle bundles (Fig. 1C–F). Figure 1A and B demonstrate that 4 weeks of tamoxifen treatment was sufficient to essentially abolish both Orai1 transcript levels and Mn^2+^ quench, respectively. Figure 1A also includes a lane with a muscle lysate from a *mdx* mouse, in which the intensity of the band of Orai1 transcript level is visually increased compared to control. This finding is in line with the increased Orai1 expression in *mdx* muscle reported previously^1^, and thus, serves as a positive control for the specificity of the assay for the detection of Orai1 transcript.

The IF experiments in EDL muscle bundles from control and iOrai1 KO mice shown in Fig. 1C–F were conducted using the same Orai1 (PA5-26378) and RyR1 (34 C) antibodies, dilutions and conditions as described in the Article. Identical gains for confocal excitation, emission and magnification were used, thus permitting a direct comparison between signals observed in the presence (control) or absence (iOrai1 KO) of Orai1. As seen in the representative images shown in Fig. 1C and D, a similar double-row of RyR1 signal (marking the position of triads) was observed in EDL fibers from both control and iOrai1 KO mice. Importantly, an intense double-row of Orai1 fluorescence that co-localizes with the RyR1 signal (a pattern identical to that reported in Fig. 3 of the Article) was only observed in fibers from control mice. Specifically, the Orai1 peak signals coincident with those of RyR1, quantified in line scans (Fig. 1E) across multiple fibers, was reduced by ~85% in fibers from iOrai1 KO mice (n = 12 fibers) compared to that observed for control fibers (n = 14 fibers) (Fig. 1F). The calculated ratio of Orai1 signal at the triad (i.e. in correspondence to the peak of RyR1 signal) to that in the A band (i.e. the signal between two consecutive double-rows of RyR1 fluorescence) was 3.45 ± 0.1 in control fibers and close to unity (1.27 ± 0.06) in fibers from iOrai1 KO mice.

These data validate the specificity of the Thermo Scientific Orai1 antibody (PA-26378) for Orai1 in IF experiments in intact skeletal muscle fibers. Importantly, these results provide further support for our conclusions that Orai1 is primarily localized in the triad in muscle fibers from non-exercised mice and that a fraction of Orai1 translocates toward the Z-line following acute exercise. We propose that this translocation of Orai1 increases STIM1-Orai1 coupling and SOCE activity within the I band region of the sarcomere needed to maintain contractile force during repetitive, high-frequency stimulation (Fig. 5 in the Article).

***Animals***. Skeletal muscle-specific, tamoxifen-inducible Orai1 KO mice (*Orai1*^*fl/fl:HSA-MCM*^) were generated as described previously (Ref 18 in the Article). Four month old male *Orai1*^*fl/fl:HSA-MCM*^ mice were provided *ad libitum* access to either normal mouse chow or tamoxifen-infused (400 mg/kg) mouse chow (Envigo, Madison, WI) for 4 weeks prior to conducting mRNA, Mn^2+^ quench and IF experiments. All animal procedures were reviewed and approved by the University Committee on Animal Resources at the University of Rochester in accordance with the Guide for the Care and Use of Laboratory Animals (National Institutes of Health, Bethesda, MD).

***RNA isolation and cDNA preparation***. TA muscles were excised and snap frozen in liquid nitrogen. RNA was isolated using Trizol reagent according to the manufacturer’s protocol (15596-018; Thermo Fisher Scientific, Waltham, MA). A total of 1 µg RNA was then treated with DNase according to manufacturer’s protocol (EN0525; Thermo Fisher Scientific, Waltham, MA).

***Transcript quantification***. Semiquantitative PCR was performed using 5′ end fluorescein (6- FAM)-labeled forward primers (Integrated DNA Technologies, Coralville, IA) on 10 ng cDNA prepared as described above. Transcript levels were quantified at the 30^th^ cycle for both loading control (α_1S_-subunit of the dihydropyridine receptor or DHPR) and Orai1 as described previously (Ref 18 in the Article). The primers used were: Cacna1s (855 bp): (forward) 5′-TCATCTTCACCCTGGAGATG-3′, (reverse) 5′-TACCCTGTGTGGCAGAACTT-3′; Orai1 (307 bp): (forward) 5′- TTTAGTGCCTGCACCACAGTGCTA-3′, (reverse) 5′-TGTGGTTGGCGACGATGACTGATT-3′.

***Mn***^***2***+^ ***quench measurements***. SOCE was assessed from the maximum rate of Mn^2+^ quench of fura-2 fluorescence as described previously (Carrell *et al*. 2016). Briefly, single FDB fibers were loaded with 5 μM fura-2 AM for 1 hr at 37 °C in a Ca^2+^-free rodent Ringer’s solution containing (in mM): 145 NaCl, 5 KCl, 1 MgCl_2_, 0.2 EGTA, pH 7.4. During fura-2 loading, fibers were simultaneously treated with a SERCA pump inhibitor cocktail consisting of 1 μM thapsigargin (TG), 15 μM cyclopiazonic acid (CPA) plus 30 μM *N*-benzyl-p-toluene sulfonamide (BTS), a skeletal muscle myosin inhibitor, in order to fully deplete SR Ca^2+^ stores and inhibit movement artifacts due to fiber contraction. Store-depleted fibers were then bathed in a Ca^2+^-free Ringer’s solution and excited at 362 nm (isobestic point of fura-2) while emission was detected at 510 nm using a DeltaRam illumination system (Photon Technology, Birmingham, NJ). After obtaining a basal rate of fura-2 quench in the absence of Mn^2+^ (R_baseline_), fibers were then exposed to 0.5 mM MnCl_2_. The maximum rate of Mn^2+^ quench of fura-2 fluorescence in the presence of Mn^2+^ (R_max_) was obtained from the slope of the fura-2 emission trace during Mn^2+^ application. SOCE activity was calculated as R_SOCE_ = R_max_ − R_baseline_ and expressed as dF/dt (counts/sec).

***IF labeling and confocal microscopy***. IF studies in EDL muscle bundles from control and iOrai1 KO mice were conducted using the same primary rabbit polyclonal Orai1 (1:20, PA5-26378, Thermo Fisher Scientific, Waltham, MA) and mouse monoclonal RyR1 (1:30, 34 C antibody, Developmental Studies Hybridoma Bank) antibodies and conditions as described in the Article. Secondary antibodies used were: a) rhodamine anti-mouse IgG (1:1000) and b) Alexa-Fluor 488 goat anti-rabbit IgG (1:500). Confocal images were acquired using an Olympus FV1000MP microscope equipped with an Olympus Uplan 60X NA 1.35 oil objective. Fluorescence image profiles were obtained from exported JPEG images using ImageJ software. Identical excitation, emission and image display values were used for all images, thus permitting direct comparison between signals observed in muscle fibers from control and iOrai1 KO mice.
