# Supplementary material for: Exercise-dependent formation of new junctions that promote STIM1-Orai1 assembly in skeletal muscle
Source: Sci Rep. 2017 Oct 27;7:14286. doi: 10.1038/s41598-017-14134-0 (PMC5660245; doi:10.1038/s41598-017-14134-0)
Supplement: Supplementary file 1 — Supplementary Information [file 41598_2017_14134_MOESM1_ESM.pdf]

SUPPLEMENTARY INFORMATION

Title:

Exercise-dependent formation of new junctions that promote STIM1-Orai1 assembly in skeletal muscle

Authors:
Simona Boncompagni*[1,2], Antonio Michelucci*[1,2], Laura Pietrangelo[1,2], Robert T. Dirksen[3], and Feliciano Protasi[1,4].
*These authors contributed equally to this work.

Affiliations:
[1]CeSI-Met - Center for Research on Ageing and Translational Medicine; Univ. G. d'Annunzio, I-66100 Chieti, Italy.
[2]DNICS - Dept. of Neuroscience, Imaging and Clinical Sciences; University G. d'Annunzio, I-66100 Chieti, Italy.
[3]Department of Pharmacology and Physiology; University of Rochester Medical Center, Rochester, NY 14642.
[4]DMSI, Dept. of Medicine and Aging Science; Univ. G. d'Annunzio, I-66100 Chieti, Italy.

SUPPLEMENTARY DISCUSSION

- STIM1 may be present at SR-TT and SR-SR interfaces. Our immunofluorescence and immunogold results indicate that the SR at the I band is enriched in STIM1 (Figs. 3 and 4; and Fig. S4). EM results indicate that small electron-dense strands are visible between SR vesicles under control conditions (Fig. 1 B, inset) and within stacks of flat-cisternae following exercise (Fig. 1 D, inset; and Fig. S5). Similar electron-dense junctional strands were described previously in cells that overexpress only STIM1[35,42], consistent with the idea that ER-ER stacks containing STIM1 molecules can form independently of the presence of Orai1[43] and/or ER depletion. Thus, we propose that the small electron-dense strands visible in EM (Fig. 1 B and D; and Figs. S2 and S5) may represent STIM1 proteins (modeled in red in Fig. 7), which become more numerous and crowded following exercise, presumably due to increased aggregation (Fig. 3 D).

Electron dense strands are present within an inter-membrane junctional space that is smaller than the average junctional gap across the CRU (or triad) that contain RyRs (8.4 ± 0.1 vs. 12.7 ± 0.3 nm; Fig. 1 G). A similar junctional gap width (8.3±0.3 nm) of ER-PM junctions was reported previously

in HeLa cells transfected with YFP-STIM1[35]. Perni et al.[42] reported a slightly larger junctional gap width (12.0 ± 2.0 nm) in HEK cells overexpressing STIM1, which is likely due to a difference in the methods used to make the measurements (i.e. measured as center-to-center distance between membranes by Perni and colleagues[42] rather than the distance between adjacent membrane edges as conducted here). Importantly, we found a significant reduction in junctional gap width within CEUs following treadmill exercise (from 8.4±0.1 to 7.4±0.1 nm, $p<0.01$; Fig. 1 G), possibly reflecting a tighter "zipping" of the junctional gap by STIM1 aggregation following exercise. However, the proposed presence of STIM1 molecules at SR-SR and SR-TT interfaces (red strands in Fig. 7; and Fig. S5) does not necessarily imply that STIM1 aggregates directly hold these membranes together, as proteins other than STIM1 could be involved in stabilizing SR-SR (or SR-TT) associations. For example, the intimate association of the SR with the TT within the triad depends on junctophilin[53] and is essentially independent of both DHPR and RyR proteins[54-55]. Similarly, CEUs formed following exercise may also be stabilized by proteins other than STIM1 and Orai1.

- Experimental evidence for STIM1 and Orai1 localization in newly formed, exercise-dependent SR-TT junctions.

Our immunofluorescence and immunogold results (Figs. 3 and 4) are consistent with the intracellular positioning of STIM1 being primarily within the free-SR under control conditions (Fig. 1 A and B) and in stacks of flat-cisternae following exercise (Fig. 1 C and D). The formation of green STIM1 *puncta* (arranged in double rows) adjacent to the triad (marked with red RyR1 fluorescence) following fatigue (Fig. 3 D) is consistent with STIM1 aggregation in newly formed SR-TT junctions in the I band following exercise (Fig. S1 B). However, our results do not rule out the presence of low-level STIM1 localization in terminal cisternae in proximity of RyR1 arrays (see also Fig. S8).

The intracellular positioning of Orai1 detected by immunofluorescence and immunogold (Figs. 3 and 4) is consistent with Orai1 being located within TTs under control conditions, as Orai1 co-

localizes with RyR1 at the triad (Fig. 3 B; and Fig. S4 A). However, following exercise, a fraction of the Orai1 signal translocates deeper into the I band in proximity to STIM1 puncta (Fig. 3 E; Fig. 4, arrow; and Fig. S4 B), likely as a result of Orai1 being present in TT extensions that elongate into the I band (Fig. 2; and Fig. S3). Orai1 translocation into the I band following exercise (Fig. 3 E; and Fig. 4, arrow) promotes increased co-localization with STIM1 (Fig. 3 F; and Fig. S4 B), thus providing the structural framework for an increased STIM1-Orai1 functional interaction. Some I band staining of Orai1 is visible under control conditions (Fig. 3 B, inset). However, it is unclear if this reflects low-levels of Orai1 in the I band at rest (see Fig. 7 E) or non-specific binding of the primary antibody.

Supplementary References

53. Takeshima, H., Komazaki, S., Nishi, M., Iino, M. & Kangawa, K. Junctophilins: a novel family of junctional membrane complex proteins. *Mol Cell* **6**, 11-22, (2000).

54. Franzini-Armstrong, C., Pincon-Raymond, M. & Rieger, F. Muscle fibers from dysgenic mouse in vivo lack a surface component of peripheral couplings. *Dev Biol* **146**, 364-376 (1991).

55. Takekura, H., Nishi, M., Noda, T., Takeshima, H. & Franzini-Armstrong, C. Abnormal junctions between surface membrane and sarcoplasmic reticulum in skeletal muscle with a mutation targeted to the ryanodine receptor. *Proc Natl Acad Sci U S A* **92**, 3381-3385 (1995).

SUPPLEMENTARY FIGURES AND LEGENDS

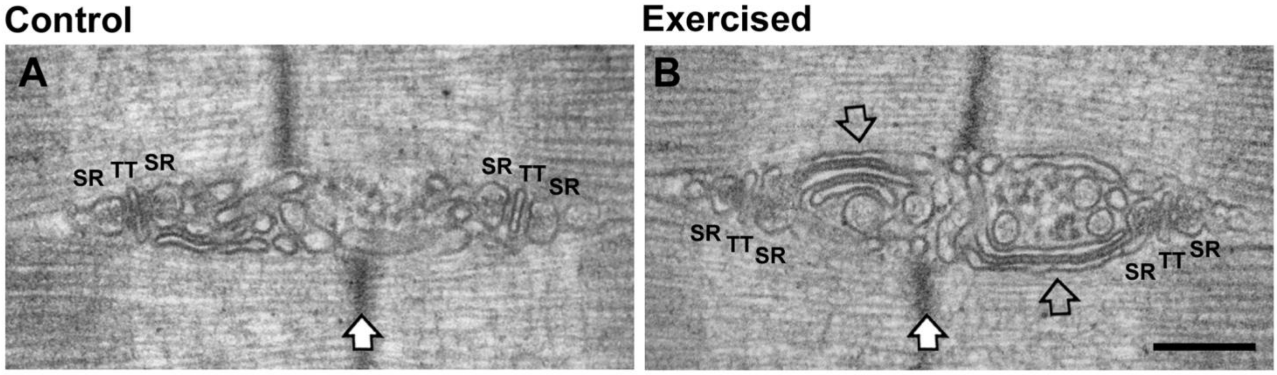

Figure S1. Remodeling of internal membranes following exercise occurs specifically within the I band. Appearance of I band membranes in EDL muscle samples from control (A) and exercised (B) mice. Empty arrows in B point to stacks of flat-parallel cisternae located between triads (labeled SR-TT-SR) and the Z lines (white arrows). Scale bar: 0.2 µm.

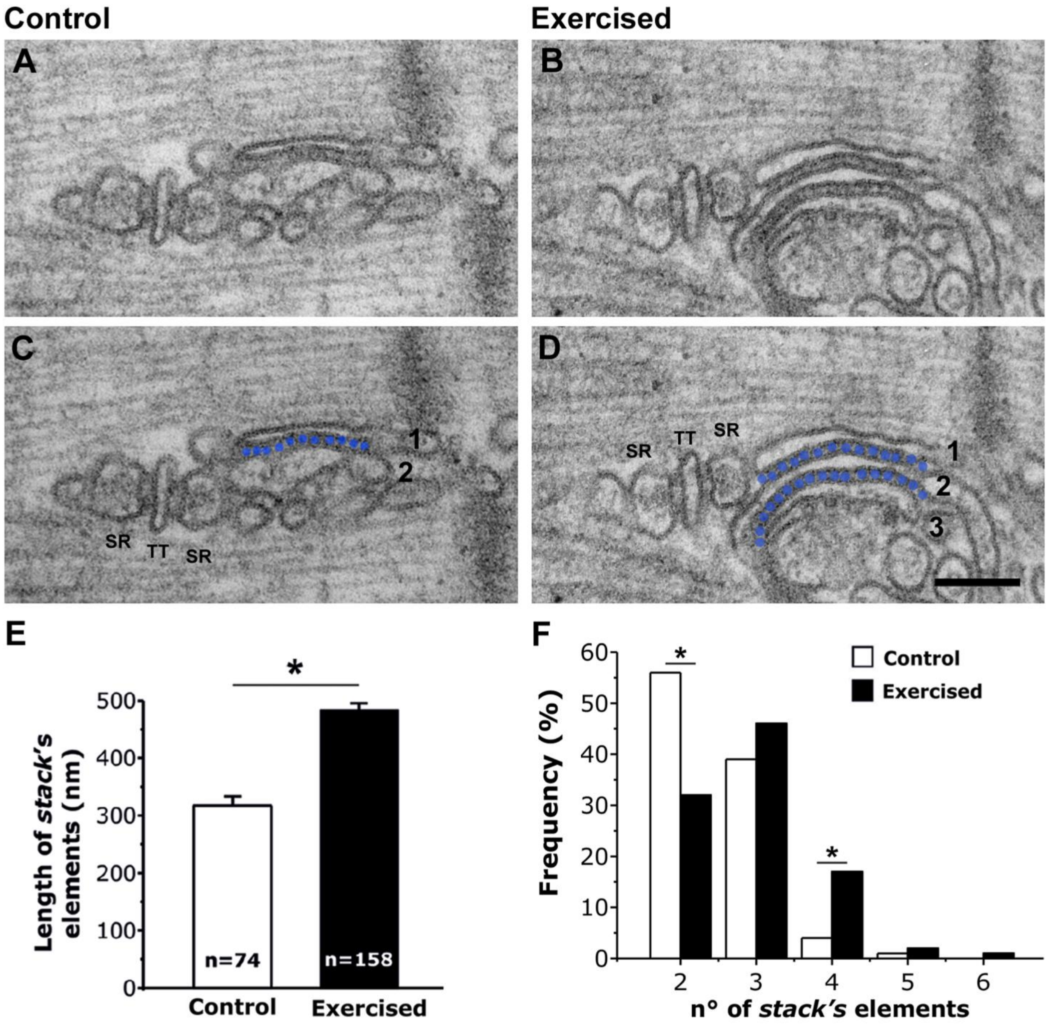

Figure S2. Stacks of membranes in muscles from exercised mice are longer and formed by more elements than those from control mice. A and B) Representative EM images of stacks of flat cisternae within the I band in EDL muscle samples from control and exercised mice. E and F) Average junctional gap length in stacks (*sample size in E: control, 3 mice/74 measurements; exercised, 3 mice/158 measurements*) and number of elements forming the stacks (*samples size in F: control, 3 mice/143 stacks analyzed; exercised, 3 mice/287 stacks analyzed*) were obtained as shown in panels C and D. In panels C and D, numbers mark each element within a stack, while dotted blue lines denote the length of the junctional gap. Data in E are shown as mean ± SEM; *$p<0.01$. Numbers in bars of panel E (n) indicate the number of stack elements analyzed. Scale bar: 0.1 µm.

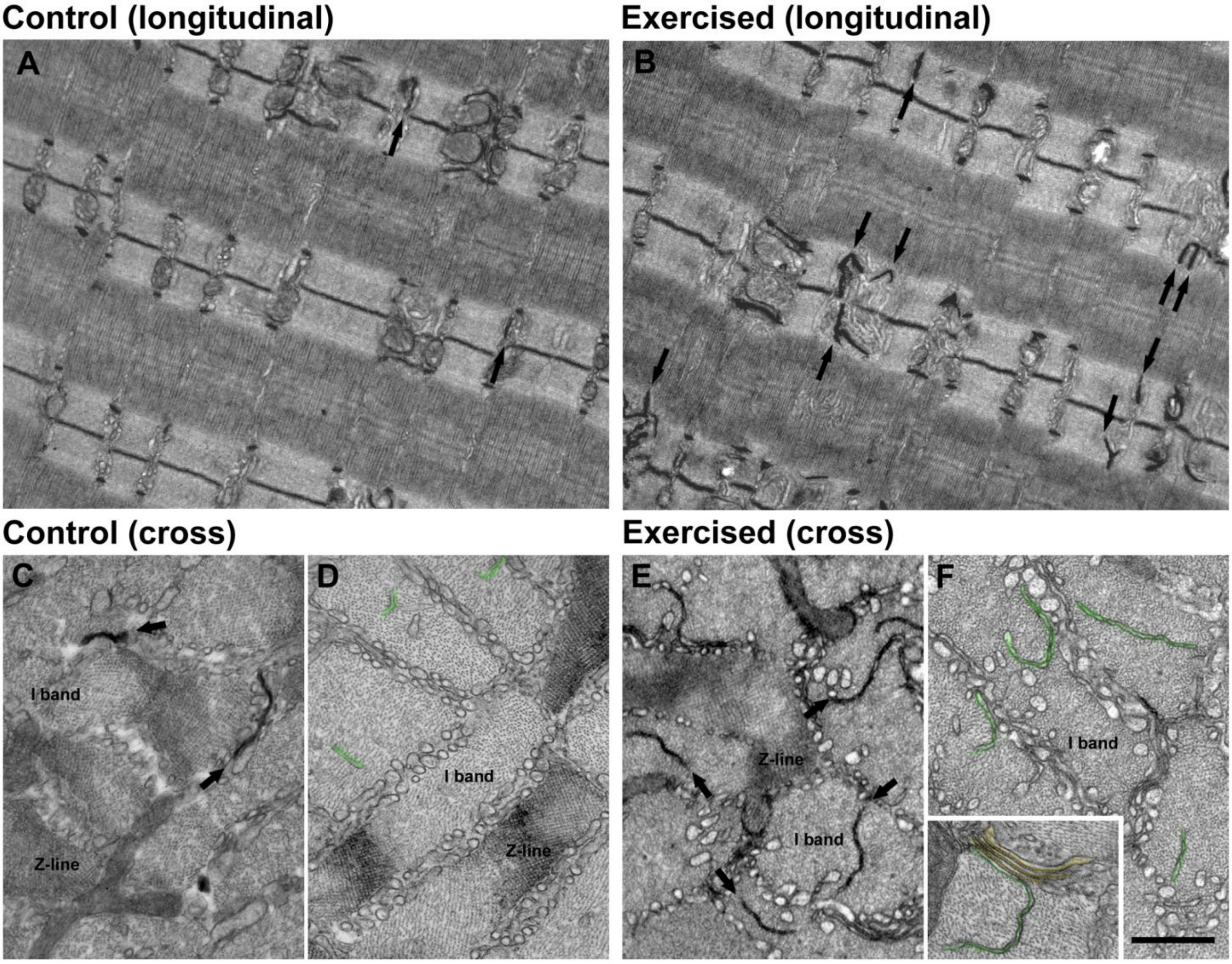

Figure S3. TTs at the I band are more frequent in EDL fibers from exercised mice. A and B) Longitudinal sections: arrows point to longitudinal TTs at the I band stained with ferrocyanide-precipitate. C-F) Cross sectional views of I band regions in EDL muscle samples from control (C and D) and exercised (E and F) mice, either stained (C and E) or not stained (D and F) with ferrocyanide. Green labels mark TTs in fibers not stained with ferrocyanide. Inset: a TT (labeled in green) associated with a stack of SR cisternae (labeled in yellow). Scale bars: A and B, 1 µm; C-F, 0.5 µm.

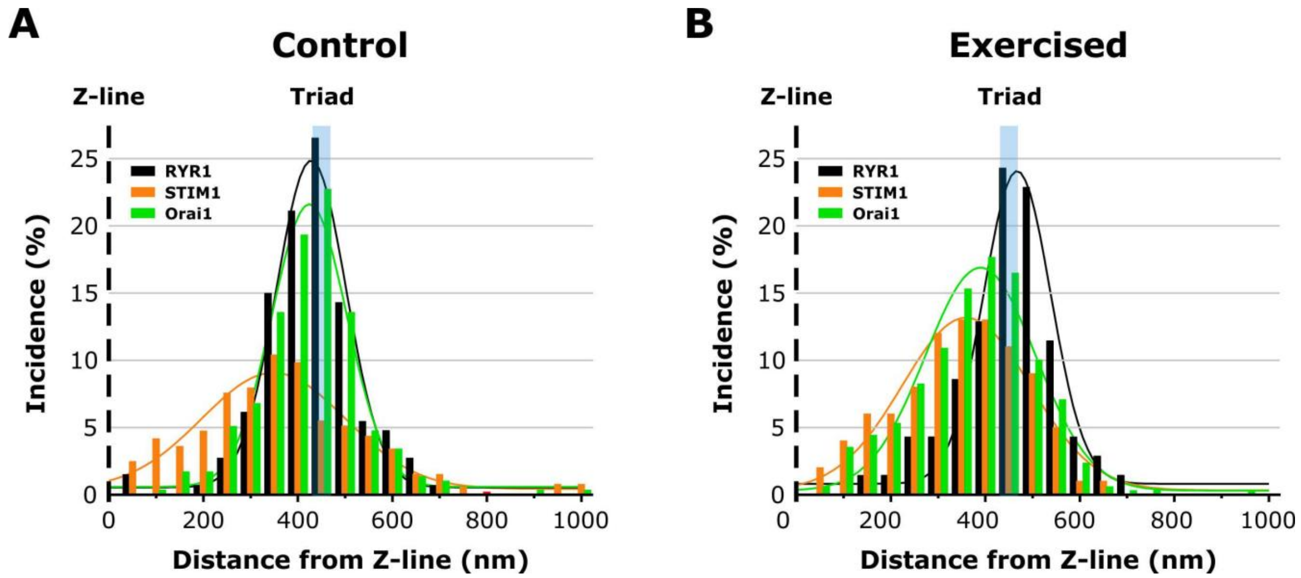

Figure S4. Following exercise, the position of immunogold particles labeling Orai1 shifts toward the Z-line. Bar graphs showing non-linear curve fit analyses of the distribution of immunogold-particles labeling RyR1 (black), STIM1 (orange) and Orai1 (green) in fibers from control (A) and exercised (B) mice. The cyan bar marks the position of the TT at the triad. A) In control fibers, STIM1 is distributed throughout the I band, with a peak ~350 nm from the Z-line, while Orai1 co-localizes with RyR1 (~450 nm from the Z-line). B) Following exercise, STIM1 and RyR1 positioning does not change, while the peak of the Orai1 distribution shifts ~100 nm toward the Z-line, resulting in increased STIM1-Orai1 co-localization. Sample size: *control, 2 mice/4-8 fibers analyzed; exercised, 3 mice/3-8 fibers analyzed.*

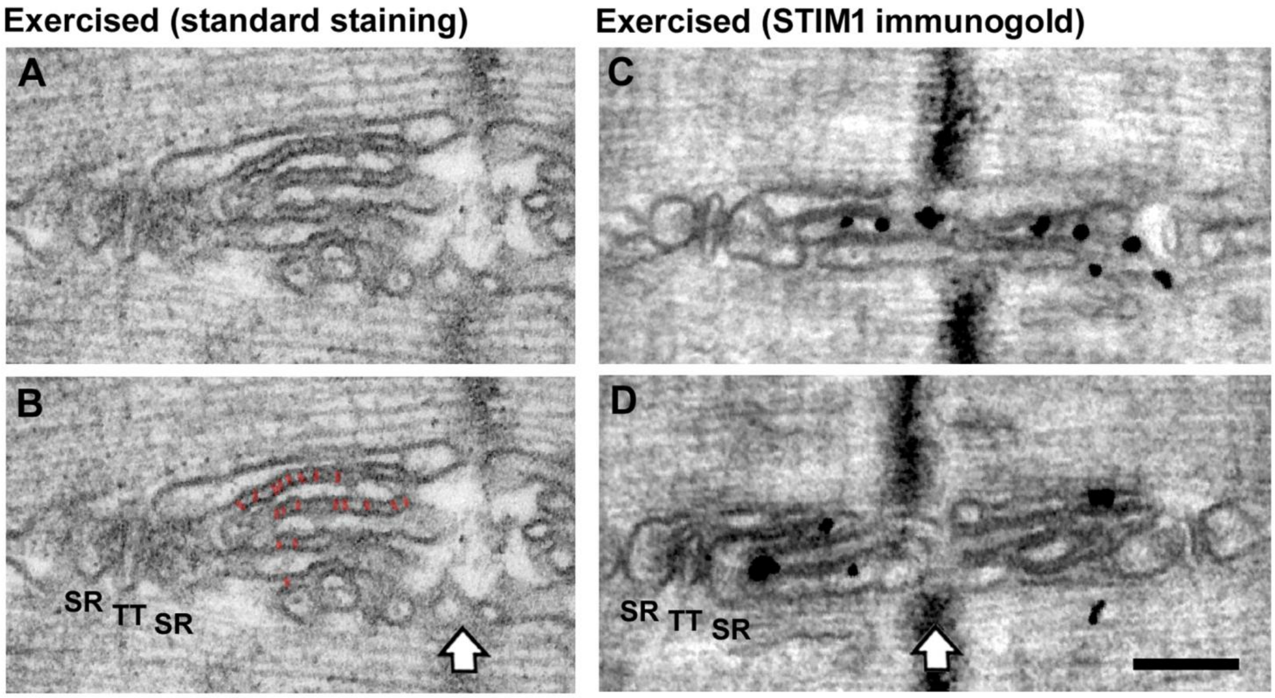

Figure S5. Stacks of flat-cisternae contain small strands coincident with STIM1 immunogold antibodies. A and B) Membrane stacks of flat-cisternae formed following exercise contain electron-dense strands (marked in red in B). C and D) Following immunogold labeling for STIM1, stacks are labeled by gold particles (black dots). In panels B and D, white arrows point to Z lines and SR-TT-SR labels indicate triads. Scale bar: 0.2 µm.

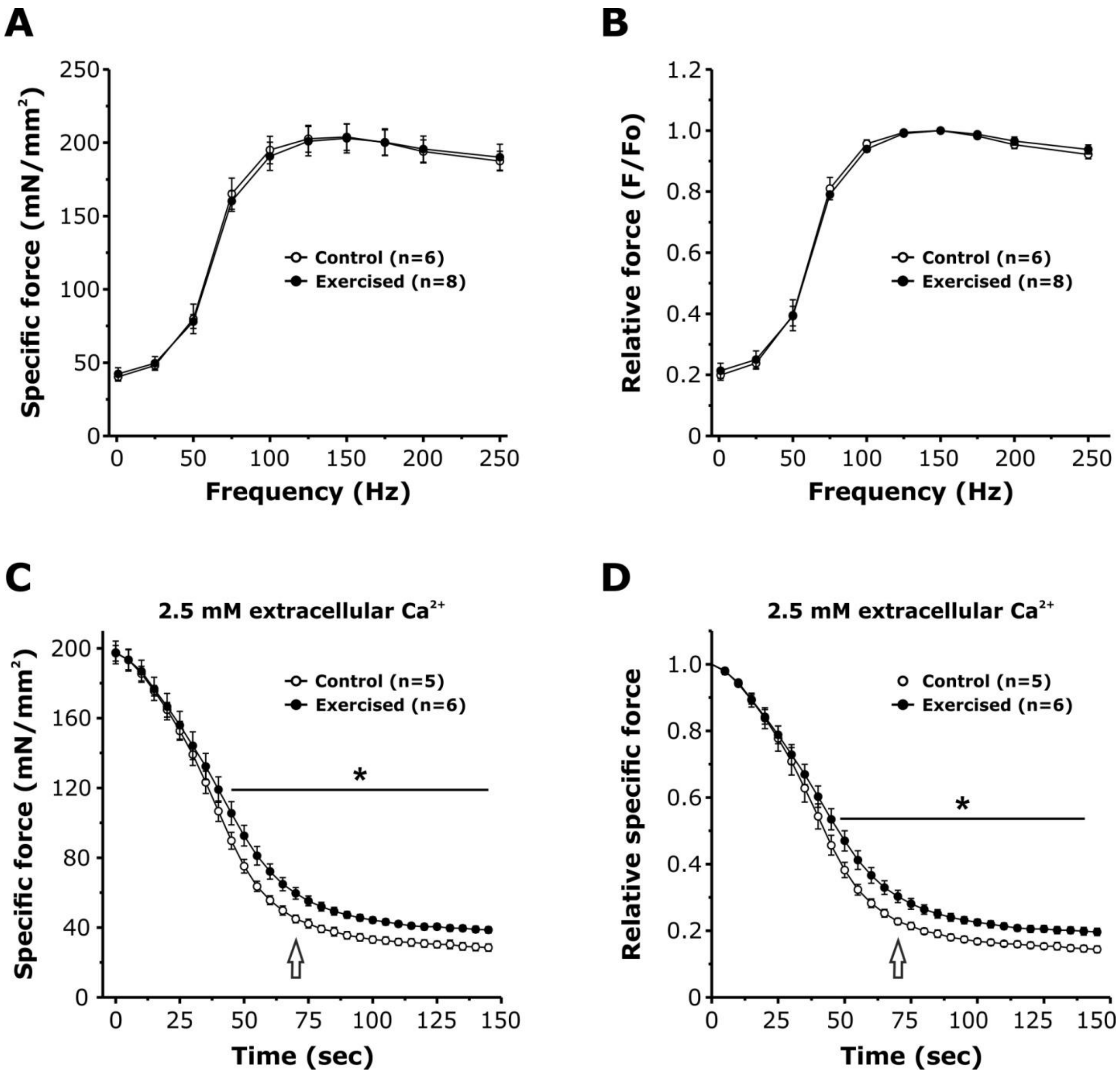

Figure S6. EDL muscles from exercised mice do not exhibit changes in force-frequency but exhibit an enhanced resistant to fatigue even at a rate of 100 Hz stimulation. A) Specific force-frequency curves obtained by applying stimulus trains from 1 to 250 Hz in EDL muscles from control and exercised WT mice using a control KH solution containing 2.5 mM Ca^{2+}. B) Relative force-frequency curves (normalized to the maximal force at 150 Hz) for the results showed in (A). C) Time course of average specific force decay during 30 consecutive high frequency stimulation trains (100 Hz, 1s duration, every 5 s). D) Time course of relative force decay (normalized to the 1st stimulus train) for the results shown in (C). All data are shown as mean ± SEM; *$p<0.05$. Number of experiments (n) reflect the number of EDL muscles analyzed.

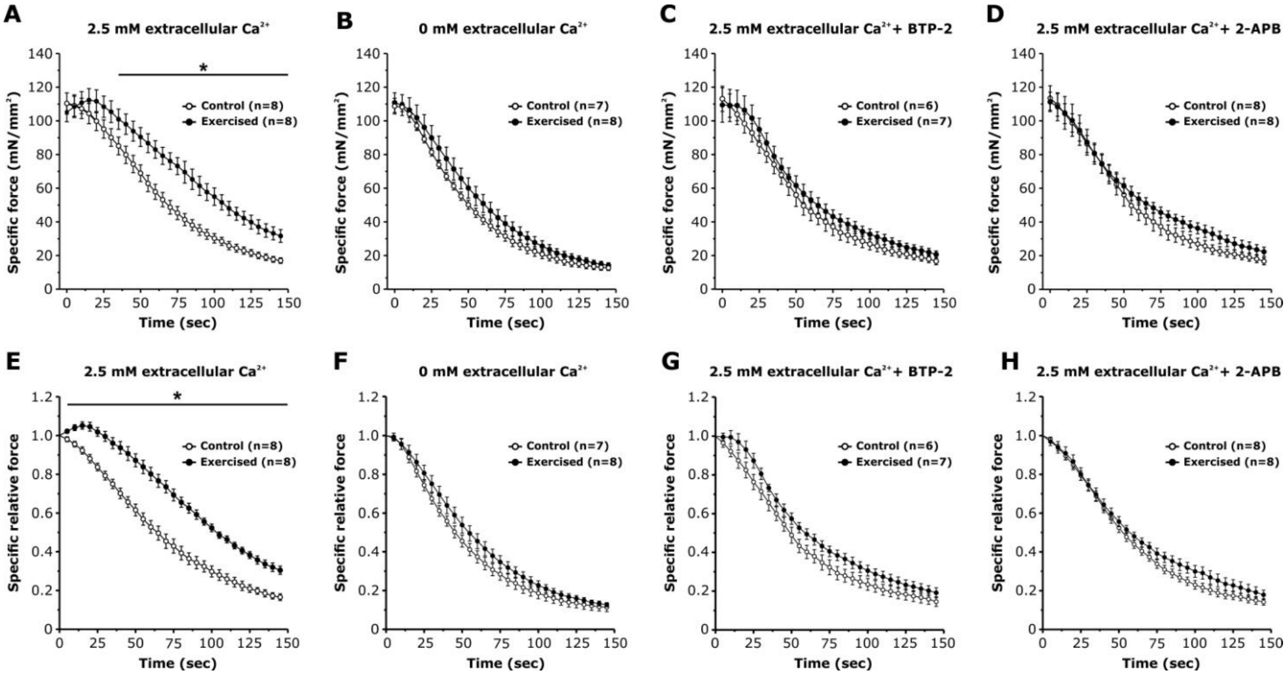

Figure S7. Dependence of enhanced resistance to fatigue of EDL muscles from exercised mice on extracellular Ca^{2+} and SOCE. Time course of peak specific (mN/mm^2) (A-D) and relative (E-H) force decay during repetitive high frequency stimulation (60Hz, 1s duration, every 5s) in EDL muscles from control and exercised mice recorded under different extracellular conditions: A and E) physiological KH solution (2.5 mM Ca^{2+}); B and F) Ca^{2+}-free KH solution (equimolar substitution with Mg^{2+}); C, D, G, and H) physiological KH solution supplemented with either 10 µM BTP-2 (C and G) or 100 µM 2-APB (D and H). Data are shown as mean ± SEM; *p<0.01. Number of experiments (n) reflects the number of EDL muscles analyzed for each condition.

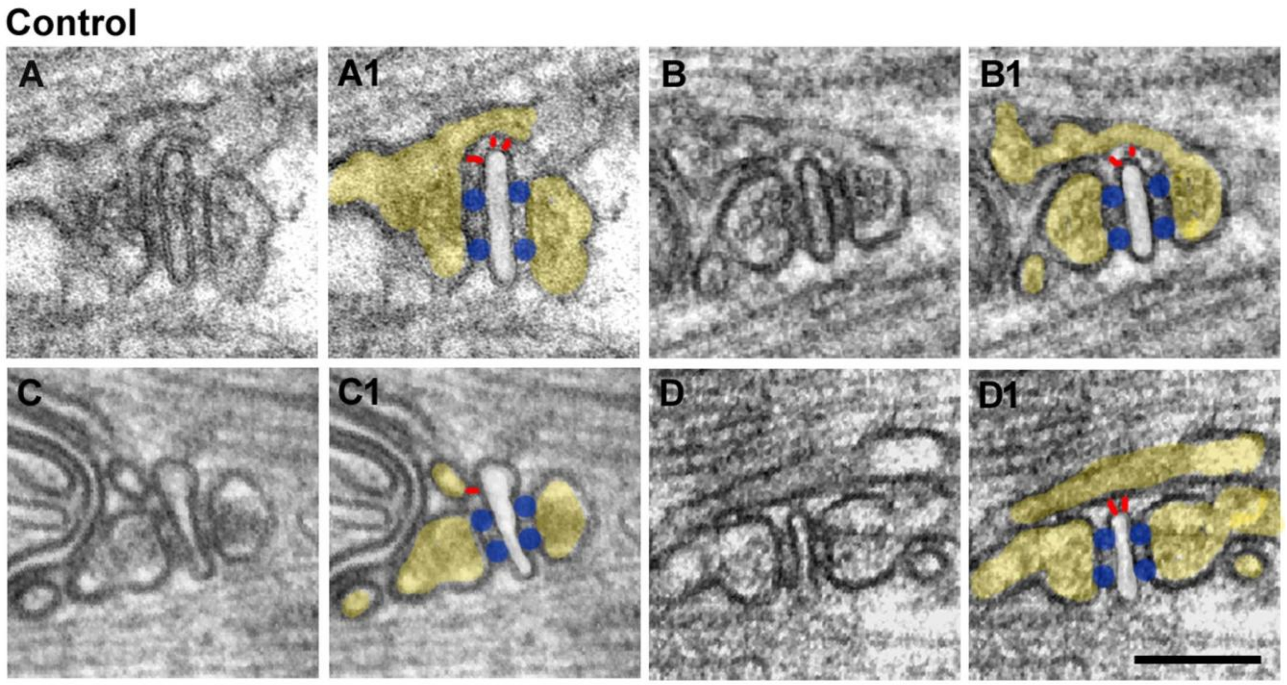

Figure S8. In control fibers, contacts between SR and TT are visible at non-junctional regions of the TT in close proximity to the triad junction. Small electron-dense strands connecting SR membranes with lateral (i.e. non-junctional) regions of TTs are sometimes visible in high resolution EM images of triads. These contacts are modeled and indicated by empty arrows in the cartoon shown in Fig. 6 A. These small electron-dense strands (labeled in red in panels A1, B1, C1, and D1) could represent aggregated STIM1 proteins. Color legend: yellow, SR; white, TT; red, STIM1; blue, RyR. Scale bar: 0.1 µm.
